# Weighing homoplasy against alternative scenarios with the help of macroevolutionary modeling: A case study on limb bones of fossorial sciuromorph rodents

**DOI:** 10.1002/ece3.5592

**Published:** 2019-09-09

**Authors:** Jan Wölfer, John A. Nyakatura

**Affiliations:** ^1^ AG Morphologie und Formengeschichte Institut für Biologie Humboldt‐Universität zu Berlin Berlin Germany; ^2^ Bild Wissen Gestaltung, Ein Interdisziplinäres Labor Humboldt‐Universität zu Berlin Berlin Germany

**Keywords:** convergence, femur, locomotion, Ornstein‐Uhlenbeck model, scapula, selective regime

## Abstract

Homoplasy is a strong indicator of a phenotypic trait's adaptive significance when it can be linked to a similar function. We assessed homoplasy in functionally relevant scapular and femoral traits of Marmotini and Xerini, two sciuromorph rodent clades that independently acquired a fossorial lifestyle from an arboreal ancestor. We studied 125 species in the scapular dataset and 123 species in the femoral dataset. Pairwise evolutionary model comparison was used to evaluate whether homoplasy of trait optima is more likely than other plausible scenarios. The most likely trend of trait evolution among all traits was assessed via likelihood scoring of all considered models. The homoplasy hypothesis could never be confirmed as the single most likely model. Regarding likelihood scoring, scapular traits most frequently did not differ among Marmotini, Xerini, and arboreal species. For the majority of femoral traits, results indicate that Marmotini, but not Xerini, evolved away from the ancestral arboreal condition. We conclude on the basis of the scapular results that the forelimbs of fossorial and arboreal sciuromorphs share mostly similar functional demands, whereas the results on the femur indicate that the hind limb morphology is less constrained, perhaps depending on the specific fossorial habitat.

## Graphical abstract

This study is concerned with the scapular and femoral trait evolution in sciuromorph rodents with an emphasis on trait homoplasy between two fossorial lineages, Marmotini and Xerini. Evolutionary model comparison does not suggest homoplasy to be a likely scenario. Instead, it appears that the scapula is more conserved, reflecting the ancestral arboreal condition, whereas the femoral morphology shifted away from this condition in Marmotini, but not in Xerini.

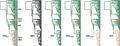

## 1 INTRODUCTION

The term homoplasy comprises all concepts that refer to two or more taxa sharing a phenotypic trait that has independently evolved similar character states (Wake, 2007; Wake, Wake, & Specht, 2011). This includes, for example, the evolution toward a former phenotypic character state of an ancestor (reversal), or the independent evolution of a novel character state on the basis of similar or different developmental genetic mechanism (parallelism and convergence, respectively; Wake et al., 2011). Homoplasy has been used as an indicator of the adaptive significance of these character states, especially when these can be associated with a similar function (Harvey & Pagel, 1991; Mayr, 1963; Nyakatura, 2012; Schluter, 2000; Simpson, 1953). Thus, it is not surprising that plenty of studies in functional morphology have been concerned with this phenomenon and aimed at unveiling particularly instructive cases of form–function relationships that evolved multiple times independently (e.g., Botton‐Divet, Cornette, Houssaye, Fabre, & Herrel, 2017; Dublin, 1903; Hildebrand & Goslow, 1995; Houssaye & Fish, 2016; Lull, 1904; Mahler, Ingram, Revell, & Losos, 2013; Moen, Irschick, & Wiens, 2013; Montañez‐Rivera, Nyakatura, & Amson, 2018; Muschick, Indermaur, & Salzburger, 2012; Osburn, 1903; Runestad & Ruff, 1995; Shimer, 1903).

Some of the most instructive examples of homoplasy stem from the limbs of tetrapods. For example, various mammalian lineages that independently acquired a fully or partly subterranean lifestyle also independently evolved a larger in‐lever to out‐lever ratio for muscles which retract the arm (teres major, and latissimus dorsi) and/or extend the forearm (triceps) as compared to cursorial relatives (Hildebrand & Goslow, 1995). This increases the muscles' force output, which was interpreted to be advantageous for digging activities (Hildebrand & Goslow, 1995). Samuels and Van Valkenburgh (2008) could demonstrate that scratch‐digging rodents in distantly related taxa also display similarities in the hind limb anatomy. According to them, one shared similarity as opposed to their terrestrial relatives constitutes a larger ratio between the height of the greater trochanter on the femur and the length of the femur and thus a larger mechanical advantage of the gluteus muscles that attach to this trochanter and retract the hind limb. This was interpreted to assist the stabilization of the body against forces produced by the forelimb during digging (Samuels & Van Valkenburgh, 2008).

These examples demonstrate that homoplasy in limb traits of fossorial mammals can be a particularly revealing topic for investigation to foster the understanding of how form reflects function. Many similar studies have been conducted on fossorial mammals or at least included taxa with independent acquisitions of a fossorial lifestyle (e.g., Carrizo, Tulli, & Abdala, 2014; Carrizo, Tulli, Dos Santos, & Abdala, 2014; Hildebrand & Goslow, 1995; Hopkins & Davis, 2009; Lehmann, 1963; Lessa, Vassallo, Verzi, & Mora, 2008; Morgan, 2009; Morgan & Álvarez, 2013; Piras et al., 2012; Samuels & Van Valkenburgh, 2008; Stein, 2000; Warburton, Grégoire, Jacques, & Flandrin, 2013). However, only a few have been put into the framework of phylogenetic comparative methods (PCMs) with a focus on homoplasy in fossorial taxa (e.g., Meier, Bickelmann, Scheyer, Koyabu, & Sánchez‐Villagra, 2013; Piras et al., 2012). One important aspect of a phylogenetic framework is to account for the directionality of evolutionary transformations (Cracraft, 1980). This is important, because examples of homoplasy can only be inferred if the supposedly homoplastic character states are indeed identified as independently evolved novel character states in all focal groups or as at least one independent acquisition of the ancestral character state in case of a reversal. In an adaptive context, this has to be equally true for form and function (Losos, 2011). PCMs represent a class of inferential statistics that utilize this phylogenetic framework to evaluate whether the species' traits within a clade of interest have evolved by chance or resulted from adaptive evolution (Felsenstein, 1985; Hansen, 1997; Harvey & Pagel, 1991; Ingram & Mahler, 2013). However, only a few PCMs have been developed to investigate the adaptive significance of homoplasy (reviewed in Stayton, 2015; and see Khabbazian, Kriebel, Rohe, & Ane, 2016 for a more recent method).

The most suitable PCM to assess homoplasy available to date, in our opinion, is the likelihood comparison among a set of macroevolutionary models, because one can compare homoplasy hypotheses to multiple alternative plausible scenarios. A frequently used model is the Ornstein–Uhlenbeck (OU) model which was introduced into a macroevolutionary context by Hansen (1997). The OU model assumes that a trait evolves toward a hypothetical optimal state θ that is best suited to accomplish the functions imposed by the selective regime with the highest selective pressure on that trait. Trait evolution is thereby driven by an adaptive rate *α* and random perturbations *σ* which prevent the species to reach the optimum as a result of less influential selective factors and historical constraints (Hansen, 1997). The Brownian motion (BM) model, which was introduced into a macroevolutionary context by Felsenstein (1985), can be regarded as a special case of the OU model with *α* being zero and hence reflecting a random walk‐like trait evolution. The likelihood of competing models can then be compared as suggested by Butler and King (2004).

A very suitable design using macroevolutionary model comparison to evaluate homoplasy in the context of adaptation is presented by Moen, Morlon, and Wiens (2015). They compared the likelihood of frog postcranial morphology reflecting either the independent acquisition of lifestyles, phylogenetic history, or a combination of both factors. They split the selective regimes (lifestyle groups) in order to evaluate the frequency of trait homoplasy on different levels of the phylogeny (homoplasy across the whole phylogeny vs. homoplasy only within biogeographical separated subclades vs. all independent lifestyle acquisitions with different optima). It needs to be emphasized that trait homoplasy in the context of OU model comparison is evaluated in respect to the trait's optima (i.e., trait optimum homoplasy) and not to the species' trait values themselves (i.e., trait homoplasy). Besides models with different levels of homoplasy, Moen et al. (2015) accounted for further plausible scenarios by including models into the comparison that represent nonadaptive evolution or evolution of all species toward a common optimum.

Here, we used this approach by Moen et al. (2015) to evaluate the likelihood of postcranial homoplasy in the limb morphology of extant sciuromorph rodent lineages that independently acquired a fossorial lifestyle. We complemented their approach by the method by Boettiger, Coop, and Ralph (2012) for measuring statistical power. We only included two of the three fossorial sciuromorph lineages, Marmotini and Xerini, because the third lineage (*Aplodontia rufa*) is monospecific and we do not consider it meaningful to estimate an optimum trait value using a single species. Marmotini is spread across the Holarctic region, and Xerini is present in Africa and Central Asia (Nowak, 1999; Thorington Jr., Koprowski, Steele, & Whatton, 2012). Species of both taxa dig underground burrows, but search for food above ground (Nowak, 1999; Thorington Jr. et al., 2012). Phylogenetic reconstructions suggest these two lineages to be monophyletic (Fabre, Hautier, Dimitrov, & Douzery, 2012; Zelditch, Li, Tran, & Swiderski, 2015), and reconstructions of ancestral lifestyles indicate independent acquisitions of a fossorial lifestyle from an arboreal ancestor (Rocha, Leite, Costa, & Rojas, 2016; Steppan, Storz, & Hoffmann, 2004; Wölfer, Amson, et al., 2019).

As homoplasy in the morphology of the skeletal limb elements of fossorial mammalian taxa has been previously demonstrated in both, fore limbs and hind limbs (see above), we compared homoplastic tendencies between the scapula and the femur, (i.e., the most proximal functional skeletal elements of therian limbs, respectively; Fischer, 1994; Fischer & Blickhan, 2006; Fischer, Schilling, Schmidt, Haarhaus, & Witte, 2002; Kuznetsov, 1985). The scapula and femur were also chosen, because they exhibit structures that allow us to derive muscle properties from the morphology of the bone. We analyzed various univariate traits reflecting length, robustness, and the ability to transmit forces by the attaching muscles. This gave us an idea about how these traits conform or differ in the most likely process of trait evolution.

The here‐investigated traits of the sciuromorph scapula and femur were recently investigated in terms of how they reflect differences in lifestyle and body mass by Wölfer, Arnold, and Nyakatura (2019) and Wölfer, Amson, et al. (2019), respectively. Both studies revealed that some traits differ significantly between the arboreal and fossorial species. In case of such a significant difference, the trait value of the fossorial group (including *A. rufa*, Marmotini, and Xerini) was always smaller than that of the arboreal group. In both cases, the authors assumed that this reflects lower demands of a fossorial lifestyle regarding running velocity (a relatively shorter femur leads to shorter limb length), bone robustness, and muscle force output. Both of these studies found the phylogenetic inertia to be low in all traits, suggesting differences to bear an adaptive signal (Wölfer, Amson, et al., 2019; Wölfer, Arnold, et al., 2019). This leads to the question as to whether this hypothesized adaptedness of proximal limb bone traits in Marmotini and Xerini is reflected in homoplasy. Especially, the differences in clade size of these two tribes render it problematic to infer homoplasy simply from a significant difference between the fossorial and arboreal groups. Marmotini with approx. 40 sampled species could have potentially dominated the outcome of the significance in comparison to Xerini with only six extant species, though all were sampled by Wölfer, Amson, et al. (2019) and Wölfer, Arnold, et al. (2019). Hypothetically, Xerini could still share an optimum with the arboreal species, while only Marmotini evolved away from their shared optimum toward a smaller trait optimum. Similarly, we also considered those traits that did not previously display a significant difference between fossorial and arboreal species, because it can be revealing about whether only Marmotini still shares an optimum with the arboreal group, with Xerini having evolved toward a different—perhaps even larger—trait optimum. Another reason for including these traits was our interest in which model would tend to determine trait evolution in case homoplasy was not clearly supported over all alternative scenarios. This gave us an idea about the frequency of each model being the most likely among all considered traits of a skeletal element.

Two previous studies (Mielke et al., 2018; Scheidt, Wölfer, & Nyakatura, 2019) analyzed further robustness parameters of the sciuromorph femur (trabecular parameter of the femoral head and cross‐sectional properties along the proximodistal axis, respectively). Mielke et al. (2018) found no significant differences between the arboreal and fossorial groups. Scheidt et al. (2019) demonstrated that fossorial sciuromorphs are more robust in the distal epiphyseal region than their arboreal relatives. We did not consider the femoral traits investigated by Mielke et al. (2018) and Scheidt et al. (2019), as only four of six xerine species were sampled in both studies what we considered too few to reliably estimate trait optima. Moreover, the dataset of Scheidt et al. (2019) is too large (i.e., 57 variables) for the computationally heavy methods applied herein.

We first explain how the functionally relevant univariate traits were acquired. This is followed by a subtraction of the statistical effect of body mass on those traits. Then, we explain the considered models and how we compare their likelihood to evaluate overall macroevolutionary patterns. We also integrated a simulation study to assess the effect of our sampling design on the likelihood comparison. Finally, in case likelihood comparison suggests a potential case of homoplasy, we utilized pairwise evolutionary model comparison sensu Boettiger et al. (2012) to assess whether the data and phylogeny are powerful enough to unambiguously support this hypothesis in favor of all other considered models.

## 2 MATERIAL AND METHODS

### 2.1 Software

Functions from the software R version 3.5.2 (R Development Core Team, 2018) were used for all analyses, including utility functions of several packages for data preparation and visualization (geomorph: Adams, Collyer, & Kaliontzopoulou, 2018; GEIGER: Harmon, Weir, Brock, Glor, & Challenger, 2008; phytools: Revell, 2012; Morpho: Schlager, 2017; tidyverse: Wickham, 2017).

### 2.2 Phylogenetic tree

We used the compound phylogenetic tree that was assembled by Wölfer, Amson, et al. (2019); displayed in their Supporting Information) as the raw, unpruned phylogeny. It consists of extant species from the maximum credibility consensus tree presented by Zelditch et al. (2015) and from the TimeTree database (Hedges, Marin, Suleski, Paymer, & Kumar, 2015).

### 2.3 Morphological traits

Our investigation is based on the scapular dataset acquired by Wölfer, Arnold, et al. (2019) and the femoral dataset acquired by Wölfer, Amson, et al. (2019). The former study used regressions to analyze 15 univariate scapular traits as well as scapular shape (i.e., a multivariate trait) in regard to an allometric effect and whether this effect differs depending on locomotor ecology (termed interaction effect). Wölfer, Amson, et al. (2019) used the same approach to study 11 univariate femoral traits as well as femoral shape. Here, we only investigated univariate traits of both skeletal elements, because likelihood estimation is unreliable when fitting complex multivariate models (Adams & Collyer, 2017).

The scapular dataset acquired by Wölfer, Arnold, et al. (2019) consisted of 186 species, and the femoral dataset acquired by Wölfer, Amson, et al. (2019) comprised 177 species represented by one specimen each with the single exception of three femoral specimens of *A. rufa* (Tables S1 and S2, respectively, in Supplementary Material). Specimens of different sex were included depending on availability (Tables S1 and S2). The level of maturity was assessed by comparing the sizes of specimens available at the collection sites and always selecting one of the larger bones. The fusion of the epiphyses was not used as criterion, as this was not observed to be the rule even in the largest specimens. Thus, a sensitivity analysis was conducted to assess the influence of sampling bias on evolutionary model comparison (see below).

According to Wölfer, Arnold, et al. (2019) and Wölfer, Amson, et al. (2019), four univariate traits of the scapula and the femur, respectively, displayed a significant interaction effect between lifestyle and body mass. This interaction effect might influence the differences among groups if these differ in their body mass. Thus, it would not be possible to discriminate between trait homoplasy between Marmotini and Xerini as a result of independent lifestyle acquisitions and trait similarity as a consequence of the interactive influence of body mass and lifestyle. We tested whether the body mass ranges of the three groups differed significantly (S1). As this was the case between each fossorial group and the arboreal group (Tables S3 and S4), we decided to only include those traits here that did not display a significantly interaction effect that resulted in a different slope between the fossorial and arboreal groups in the previous studies by Wölfer, Arnold, et al. (2019) and Wölfer, Amson, et al. (2019).

The univariate traits analyzed here cover various aspects of the scapula and the femur, such as the effective length, the robustness of the articulation sites, and the muscle properties that can be derived from the morphology of the bone (the sizes of the attachment sites as well as the lengths of the in‐levers). They will be briefly described in the following, but see Wölfer, Arnold, et al. (2019) and Wölfer, Amson, et al. (2019) for a detailed description of the acquisition of those traits.

**Figure 1:**
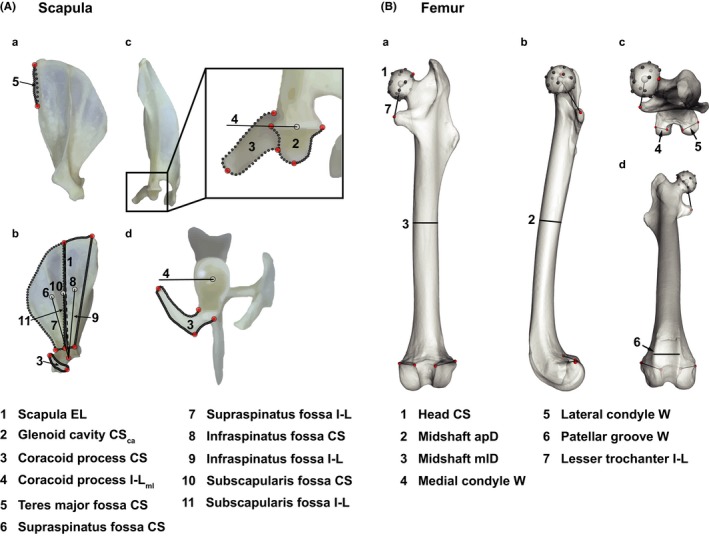
Investigated traits of the scapula and femur. A: Lateral (a), medial (b), caudal (c), and ventral (d) views on the scapula. Red circles = fixed landmarks. Dark gray circles = curve landmarks that were evenly spaced between the fixed landmarks to capture the outlines of the scapula. Light gray circles = either centroids of traits 6, 8, and 10 (b) or points used to compute trait 4 (c, d). B: Caudal (a), medial (b), dorsocaudal (c), and ventrocranial (d) views on the femur. Red circles = fixed landmarks. Dark gray circles = sliding semi‐landmarks. Light gray circle = centroid to compute trait 1. apD, anteroposterior diameter; ca, caudal view; CS, centroid size; EL, effective length; I‐L, in‐lever length; ml, mediolateral; mlD, mediolateral diameter; W, width

The scapular traits were extracted from landmarks that were placed onto images obtained from four different perspectives orthogonal to each other (lateral, medial, caudal, and ventral; Figure 1; see Wölfer, Arnold, et al., 2019). Wölfer, Arnold, et al. (2019) defined the effective length of the scapula as the distance between the approximated proximal and distal centers of rotation from the medial perspective (the point where the spine leads into the vertebral margin to the center of the glenoid cavity). The scapular robustness measures concerned the glenoid cavity (articulates with the humerus) and the coracoid process (articulates with the clavicula). The size of the glenoid cavity was measured from the caudal perspectives by using the surrounding landmarks to compute its centroid size (i.e., the spread around its mean). The size of the coracoid process was measured from the medial, caudal, and ventral perspectives. Each time, the landmarks surrounding it were used to obtain its centroid size; summing all three sizes yielded an overall estimate of the coracoid process' robustness. The remaining scapular traits related to the force transmission properties of the attaching muscles. The length of the mediolateral in‐lever of the muscles attaching to the tip of the coracoid process (short head of the biceps brachii and coracobrachialis) determines their potential to create adduction forces. It was measured from the mediolateral center of the glenoid cavity to the process' tip along the mediolateral axis of the scapula. Its value was obtained from the caudal and ventral perspectives and averaged. The sizes of the attachment areas of the short biceps and coracobrachialis muscles were not accounted for, because the muscles' attachments are tendinous and not fleshy (Bezuidenhout & Evans, 2005; Brizzie, 1941) with the biceps sometimes attaching to the coracobrachialis muscle (Parsons, 1894). The teres major muscle acts as an arm retractor by flexing the shoulder joint. Its fossa on the scapula was surrounded with landmarks from the lateral perspective to measure its centroid size. The length of the muscle's in‐lever was not obtained as the glenoid cavity was rarely visible from the lateral perspective. The rotator cuff muscles (supraspinatus, infraspinatus, and subscapularis) have fleshy attachments and stabilize the shoulder by inserting on the humeral head, close to the center of rotation. The supraspinatus and infraspinatus muscles attach on the lateral, the subscapularis muscle on the medial surface of the scapula. However, all considered traits were measured from the medial perspective as the scapula approximates a flat structure and the glenoid cavity was always visible from the medial perspective. These traits were the centroid sizes of their muscle attachment sites and the lengths of their in‐levers from the centroids to the center of the glenoid cavity.

The femoral traits were obtained by Wölfer, Amson, et al. (2019) from 3D surface models. Of those that were used here, four were derived from landmark data and three were measured directly on the surface scans (Figure 1). Five robustness traits were included. The centroid size of the head was computed using the landmarks surrounding it. The anteroposterior and mediolateral midshaft diameters and the width of the patellar groove were measured directly on the surface model. The widths of the medial and lateral condyles were computed as the distance of the respective most medial and most lateral fixed landmark on the proximal site of each condyle. The only in‐lever that did not display an interaction effect concerned the muscles attaching to the lesser trochanter (most importantly the iliopsoas muscle that protracts and adducts the hind limb by flexing the hip joint).

We used the unpruned datasets (186 and 177 species for the scapula and femur, respectively) to remove trait differences that were statistically linked with differences in body mass. Body mass information was taken from the literature (Tables S1 and S2). The *lm.rrpp* function of the package “RRPP” (Collyer & Adams, 2018) with the option SS.type = “I” and 10,000 rounds of permutation was applied to obtain the residuals for the natural log‐transformed traits on natural log‐transformed body mass. We did not use phylogenetic correction for the estimation of the regression parameters, as it was shown before that phylogenetic inertia is negligible in the traits studied here (Wölfer, Amson, et al., 2019; Wölfer, Arnold, et al., 2019).

The residual datasets were pruned to match the species composition of the pruned phylogeny. Regarding the scapula, 76 arboreal species (representing the monophyletic groups Callosciurinae, Gliridae, Protoxerini, *Ratufa*, Sciurini, *Sciurotamias*, and Tamiini), 43 marmotine species, and all 6 extant species of Xerini were investigated (i.e., 125 species overall; Table S1; Figure A1). For the femur, we were able to include 74 arboreal, 43 marmotine, and the six xerine species (i.e., 123 species overall; Table S2; Figure A1). Violin plots including the arithmetic mean were generated to provide a graphical comparison of the distributions of the residuals among the three groups.

### 2.4 Model definition

**Figure 2:**
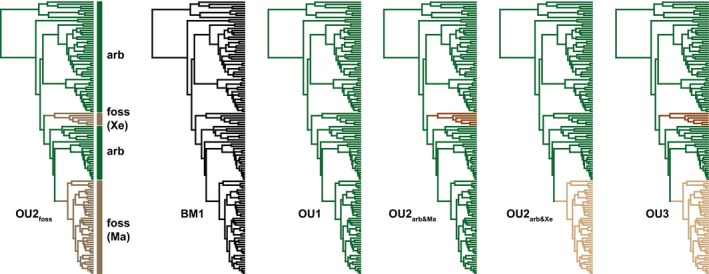
Evolutionary models compared in this study. The phylogeny of the scapular dataset was used for depiction, as the species samples for both skeletal elements were very similar. Arb, arboreal; BM, Brownian motion; foss, fossorial; Ma, Marmotini; OU, Ornstein‐Uhlenbeck; Xe, Xerini. See text for more details

The OU2_foss_ model represents the hypothesis of trait optimum homoplasy between the two fossorial clades, Marmotini and Xerini (Figure 2). In the OU2_foss_ model, both groups were expected to share the same optimum, which departed from the ancestral arboreal optimum. Five alternative models were defined. One alternative scenario was the random evolution irrespective of locomotor ecology, thus including no optima. This was represented by the Brownian motion (BM1) model of random walk‐like trait evolution with a single nonadaptive evolutionary rate. The OU1 model represented the idea that all species share the same optimum, suggesting that Marmotini and Xerini still evolve toward the ancestral arboreal optimum. The model OU2_arb&Ma_ indicated that Marmotini shares an optimum with the arboreal group, whereas the optimum of Xerini departed from this ancestral character state. The OU2_arb&Xe_ switched this assumption, assuming that Xerini shares an optimum with the arboreal species as opposed to Marmotini. The OU3 model finally represented the hypothesis that both, Marmotini and Xerini, departed from the arboreal trait optimum without sharing the same optimum. This departure could have occurred in the same direction or into different directions. In the former case, the optima of Marmotini and Xerini could have been either smaller or larger than the value of the ancestral arboreal optimum, but to a different magnitude.

### 2.5 Ancestral state reconstruction

The selective regimes had to be reconstructed on the two pruned trees for four of the six investigated models in order to evaluate their likelihood (OU2_foss_, OU2_arb&Ma_, OU2_arb&Xe_, OU3; Figure 2). For all four models, we assumed specific changes in selective regimes to occur at the root of the stem lineage leading to Xerini and/or Marmotini (Figure 2). This assumption is common for the method of “tree painting” proposed by Butler and King (2004). The *paintSubTree and paintBranches* functions of the package “phytools” (Revell, 2012) were used to define the regimes.

### 2.6 Evolutionary model comparison via likelihood scoring

We compared the likelihood of all six models for each trait using the Schwarz information criterion (SIC) that penalizes the likelihood of a model with the number of parameters estimated and the number of species included (Burnham & Anderson, 2002). Lower SIC values were interpreted as higher model likelihood (Burnham & Anderson, 2002).

The R package “mvMORPH” (Clavel, Escarguel, Merceron, & Poisot, 2015) was used to fit the OU models and the BM model with the functions *mvOU* and *mvBM*, respectively. We applied the rectangular full‐packed format algorithm option for the OU models and the phylogenetic independent contrast option for the BM1 model, respectively, to estimate the log‐likelihood of the models. The parameters and likelihood values were saved for the pairwise model comparisons (see below) and the SIC values were computed. The estimated *α* parameters were transformed into the so‐called phylogenetic half‐life *t*_1/2_ (Tables S5 and S6). This is defined as half the evolutionary time necessary for a lineage to switch to another optimum when entering a novel selective regime (Hansen, 1997) and was used to discuss historical constraints on trait evolution.

We combined the SIC scoring with a sampling simulation to evaluate whether the sampling of a single individual per species affects the outcome of the ranking (S2). In short, we assessed the intraspecific standard deviation of selected species and used them to generate hypothetical populations for all species. From those, we draw samples including a single specimen per species 1,000 times and redid the analysis each time. Finally, it was compared whether the model with the highest frequency of displaying the lowest SIC score among the simulated datasets was also the model that was demonstrated to be the most likely for the original dataset. If this was not the case, the trait was dismissed from further analysis. Accordingly, two traits were removed (the centroid sizes of the teres major and supraspinatus fossae; see Tables 1 and 2). Furthermore, we decided that it is worth continuing with the pairwise model comparison only in case the homoplasy hypothesis was suggested as the most likely model or, if not, ranked second behind the OU3 model (the only model that is more complex than the OU2_foss_ model) according to SIC. Otherwise, we did not expect the pairwise model comparison to favor the OU2_foss_ model over all other models by any chance. All traits were omitted for which this was not the case.

**Table 1:** Likelihood scoring and reliability of likelihood comparison concerning the scapular dataset

Trait	Schwartz information criterion											
	Value of empirical dataset						Frequency of lowest value among simulated datasets (%)					
	OU2_foss_	BM1	OU1	OU2_arb&Ma_	OU2_arb&Xe_	OU3	OU2_foss_	BM1	OU1	OU2_arb&Ma_	OU2_arb&Xe_	OU3
Scapula EL	−201.053	−53.719	**−204.421**	−199.679	−201.361	−196.541	0.8		**97.4**		1.8	
Glenoid cavity CS_ca_	−215.809	−74.177	**−218.387**	−214.363	−214.801	−211.335	4.6		**93.6**	1.4	0.4	
Coracoid process CS	**−39.729**	1.087	−27.855	−31.161	−26.137	−37.419	**88.1**			0.6	11.3	
Coracoid process I‐L_ml_	**−107.861**	−45.703	−87.929	−87.289	−90.449	−103.727	**99.9**					0.1
Teres major fossa CS	**170.113**	281.615	220.149	224.911	172.179	172.587	44.1		1.5		**57.4**	
Supraspinatus fossa CS	−173.421	−43.235	−174.197	−170.193	**−175.189**	−170.537	1.6		**61.0**	2.0	37.2	
Supraspinatus fossa I‐L	−210.509	−59.985	**−214.281**	−209.473	−210.417	−205.703	0.6		**99.1**	1.0	0.2	
Infraspinatus fossa CS	−189.185	−46.767	−186.635	−181.851	**−189.665**	−185.023	24.8		33.9		**41.3**	
Infraspinatus fossa I‐L	−205.687	−62.941	**−208.963**	−204.231	−206.025	−201.205	11.0		**96.4**		2.5	
Subscapularis fossa CS	−184.281	−45.941	−183.193	−178.627	**−185.429**	−180.613	8.6		43.8		**47.6**	
Subscapularis fossa I‐L	−208.643	−60.049	**−212.449**	−207.637	−208.719	−203.919	0.7		**99.0**		0.3	

Note

Value in bold: lowest Schwartz information criterion score per trait (empirical dataset) or highest frequency (simulated datasets).

See Figure 1 for trait abbreviations and text for explanation of model abbreviations.

**Table 2:** Likelihood scoring and reliability of likelihood comparison concerning the femoral dataset

Trait	Schwartz information criterion											
	Value of empirical dataset						Frequency of lowest value among simulated datasets (%)					
	OU2foss	BM1	OU1	OU2arb&Ma	OU2arb&Xe	OU3	OU2foss	BM1	OU1	OU2arb&Ma	OU2arb&Xe	OU3
Head CS	−230.851	−82.362	−220.525	−215.749	**−231.029**	−226.997	46.5		4.6		**48.9**	
Midshaft apD	−156.435	−54.388	−147.111	−143.445	**−161.979**	−157.329	1.0		0.1		**98.8**	0.1
Midshaft mlD	−145.735	−68.870	−144.283	−140.337	**−148.651**	−144.053	2.4		21.8		**75.8**	
Medial condyle W	−172.503	−31.808	**−176.799**	−174.821	−171.989	−170.075	0.5		**91.2**	8.2	1.0	
Lateral condyle W	**−179.473**	−105.684	−164.589	−160.153	−178.925	−175.873	**51.9**		0.7		47.4	
Patellar groove W	−146.473	−22.604	−125.731	−121.999	**−154.533**	−149.753	0.1				**99.9**	
Lesser Trochanter I‐L	−153.189	−11.202	−150.685	−145.883	**−153.893**	−149.223	21.6		30.4		**48.0**	

Note

Value in bold: lowest Schwartz information criterion score per trait (empirical dataset) or highest frequency (simulated datasets).

See Figure 1 for trait abbreviations and text for explanation of model abbreviations.

### 2.7 Pairwise evolutionary model comparison

Using the method developed by Boettiger et al. (2012), we assessed in a pairwise manner if the data distribution of the residuals of the remaining traits across species and the structure of the phylogenetic tree are powerful enough to discriminate between the likelihood of the OU2_foss_ model and each of the other five models.

**Figure 3:**
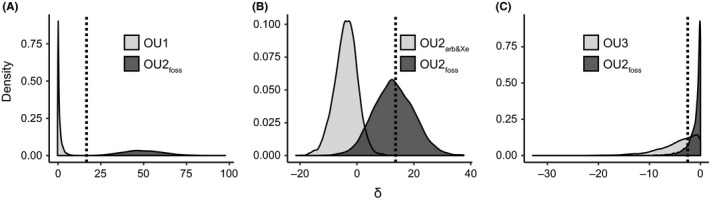
Different examples illustrating the empirical likelihood ratio in relation to simulated likelihood ratio distributions. Three examples chosen from the mediolateral length of the in‐lever of the muscles attaching to the coracoid process. The value of the empirical likelihood ratio *δ*_emp_ (dotted line) relative to the simulated distribution of the respective alternative model (A: OU1, B: OU2_arb&Xe_, C: OU3) and the simulated distribution of the homoplasy hypothesis (OU2_foss_) differs among the three examples. *δ*_emp_ either falls outside of the 95% confidence interval (CI) of both models (A), only within the 95% CI of OU2_foss_(B), or within the 95% CI of both models (C)

For each pairwise model comparison (OU2_foss_ vs. one of the five alternative models), the following procedure was applied. Firstly, the saved likelihood values of the model fits outlined above were used to compute the likelihood ratio between both models, an expression of the difference between the two likelihood values of the two models. Here, we refer to it as the empirical likelihood ratio *δ*_emp_. Secondly, the saved parameters of the two models were used to simulate two distributions of data (5,000 datasets) along the phylogenetic tree that could have evolved according to the two models. This ensures that the structure of the phylogenetic tree is accounted for when assessing the likelihood between the two models. For this purpose, the function *mvSim* of the package “mvMORPH” was used. Finally, both models were fit to all datasets of both distributions using the functions and options outlined for the evolutionary model comparison via likelihood scoring. This resulted in four distributions of parameter and likelihood values. A likelihood ratio distribution was computed for the two likelihood distributions that were fit to the same simulated data distribution. This lead to two likelihood ratio distributions (one belonging to each of the two models) and allowed us to evaluate which of the two likelihood ratio distributions better reflects *δ*_emp_ and hence which of the two models better fits the data. According to Boettiger et al. (2012), one way to do so is to check whether *δ*_emp_ lies within the 95% confidence interval (CI) of the respective likelihood ratio distribution. We encountered three scenarios: either *δ*_emp_ lay only inside the 95% CI of the distribution generated from the OU2_foss_ model, inside the 95% CIs of the distributions generated from both models, or outside of the 95% CIs of the distributions generated from both models (exemplified in Figure 3). Only the first‐mentioned case (Figure 3B) would have indicated that the distribution of the residuals across species and the structure of the phylogenetic tree are powerful enough to discriminate between the likelihood of the two models. The latter two cases would have not allowed us to discriminate between the two models. Thus, we decided that the homoplasy hypothesis can only be considered favorable in the first case. The parameter value distributions of each of the six models fit to the data simulated under the same model were used to obtain the CIs of the estimated parameters of all original model fits during evolutionary model comparison via likelihood scoring (Tables S5 and S6).

## 3 RESULTS

### 3.1 Evolutionary model comparison via likelihood scoring

Only three of six models (OU1, OU2_foss_, or OU2_arb&Xe_) scored lowest according to the SIC (Tables 1 and 2) for any trait. All estimated model parameters were reliable (i.e., they fell within their 95% CI), except for the nonadaptive evolutionary rate *σ* concerning the BM1 model (Tables S5 and S6). This rate was not reliably estimated for any trait that we studied. The largest estimate for *t*_1/2_ among all traits and all OU models fit to them was 9.5 million years. This means that the switch from one optimum to another took at most ~19 million years. According to our ancestral lifestyle reconstruction, the time the marmotine ancestor switched from an arboreal to a fossorial lifestyle was ~24.5 million years ago and that for the xerine ancestor ~31.3 million years ago.

Regarding the scapular traits, the OU1 model most frequently yielded the lowest SIC values (Table 1). This was the case for the effective length, the size of the glenoid cavity from the caudal view, and the lengths of the in‐levers of the rotator cuff muscles. The OU2_foss_ model displayed the lowest SIC score for the size of the coracoid process and the mediolateral in‐lever of the muscles attaching to this process' tip. The OU2_arb&Xe_ model yielded the lowest SIC score for the two centroid sizes of the infraspinatus and subscapularis muscle attachment sites.

**Figure 4:**
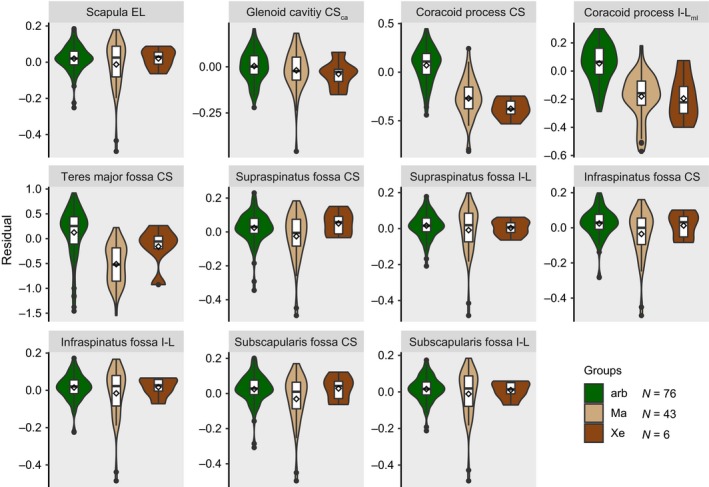
Violin plots representing the distribution of scapular trait residuals among groups. Boxplots including a density distribution are displayed for each trait (see Figure 1 for trait abbreviations) and each of the three groups (arb, arboreal; Ma, Marmotini; Xe, Xerini). A rhombus represents the arithmetic mean value. *N* = number of sampled species

**Figure 5:**
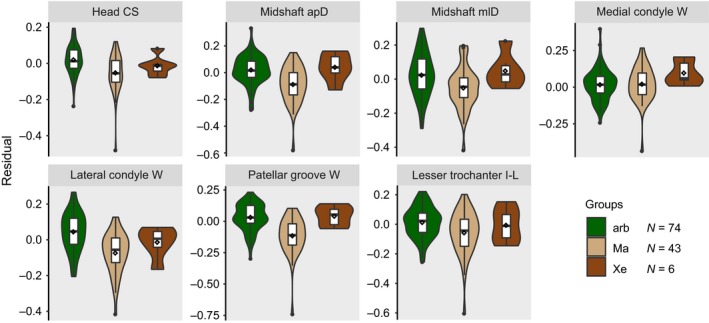
Violin plots representing the distribution of femoral trait residuals among groups. Boxplots including a density distribution are displayed for each trait (see Figure 1 for trait abbreviations) and each of the three groups (arb, arboreal; Ma, Marmotini; Xe, Xerini). A rhombus represents the arithmetic mean value. *N* = number of sampled species

Regarding the femoral traits, the OU2_arb&Xe_ model most frequently yielded the lowest SIC values (Table 2). This was the case for the centroid size of the head, the two midshaft diameters, the width of the patellar groove, and the in‐lever of the muscles attaching to the lesser trochanter. The OU1 model was the most likely one for the width of the medial condyle, and the OU2_foss_ model was the most likely choice for the width of the lateral condyle. The violin plots appear to reflect the modeling results (Figures 4 and 5).

### 3.2 Pairwise evolutionary model comparison

According to the previous steps of the analysis, only two scapular traits (the centroid size of the coracoid process, the mediolateral in‐lever of the muscles attaching to the process' tip) and one femoral trait (the width of the lateral condyle) were qualified for the investigation using the power analysis suggested by Boettiger et al. (2012). For none of these three traits, the suggested hypothesis of trait optimum homoplasy (OU2_foss_) was favoured over all the other models of trait evolution, that is, at least one alternative model could not be discriminated from the homoplasy hypothesis in terms of model likelihood (Table 3).

**Table 3:** Pairwise model comparison

Trait	OU2_foss_ versus				
	BM1	OU1	OU2_arb&Ma_	OU2_arb&Xe_	OU3
Scapula					
Coracoid process CS	/	/	/	+	=
Coracoid process I‐L_ml_	+	/	/	+	=
Femur					
Lateral condyle W	+	+	+	=	=

Note

OU2_foss_ favored over (+), equally plausible as (=), or equally implausible as (/) the respective model.

See Figure 1 for trait abbreviations and text for explanation of model abbreviations.

## 4 DISCUSSION

### 4.1 Lack of homoplasy in the sciuromorph scapula and femur

The purpose of this study was to test via macroevolutionary model comparison (a) whether two sciuromorph lineages that independently acquired a fossorial lifestyle display homoplasy in optima of functionally significant scapular and femoral traits and (b) which scenario tended to reflect trait evolution in case homoplasy was not clearly supported over all alternative scenarios. The most proximal skeletal elements of the fore limbs and hind limbs were selected in order to assess whether they display similar homoplastic tendencies, perhaps due to a similar functional constraint from a fossorial lifestyle. After a step‐wise likelihood comparison via SIC scoring and pairwise model comparison sensu Boettiger et al. (2012), the hypothesis of homoplasy was never confirmed as the single most likely model. Our study exemplifies the importance to apply power analyses with the data and tree structure. Otherwise, if we would have exclusively relied on likelihood comparison via SIC scoring, the homoplasy hypothesis would have been unjustifiably identified as the single most likely scenario for three traits (Table 3). Nevertheless, even when only considering the SIC results without the power analysis, the homoplasy hypothesis turned out as very unlikely, as it was only supported for three out of 16 traits in this step of our overall analysis.

### 4.2 Historical constraints on homoplasy

It has been noted by previous authors that the skeletal morphology of sciuromorph clades is much conserved (Black, 1963; Bryant, 1945; Cardini, Hoffmann, & Thorington, 2005; Cardini & O'Higgins, 2004; Emry & Thorington Jr., 1982; Moore, 1959) or reflecting phylogenic relationships rather than ecological differences (Cardini, 2003). In our study, the role of historical constraints limiting the realization of homoplastic trait optima can be generally questioned on the basis of the low likelihood of the BM1 model for all traits (Tables 1 and 2; also reflected in the bad estimation of *σ* according to the CIs) and the estimated phylogenetic half‐life *t*_1/2_ for all OU models (assuming that at least one OU model considered in our study is somewhat representative of the true underlying evolutionary process). The longest switch from one optimum to another took at most ~19 million years among all traits and all OU models, which is shorter than the time since Marmotini and Xerini acquired a fossorial lifestyle. Thus, all three groups (including the arboreal group, as it is the oldest one) should have already reached their optimum, independent of which OU model was most likely. Consequently, we have to assume that adaptation has played a major role in the evolution of the proximal limb elements, but did not result in homoplasy. Our sampling method (one individual per species) did only bias the modeling outcome of two traits that we consequently dismissed from further consideration. Still, for the three traits that qualified for the power analysis, the number of species per group might have favored the rejection of the homoplasy hypothesis. Although we already sampled all six extant xerine species, this number might still be too low to statisitcally assess the similarity between their trait optima on those of Marmotini. Including extinct species, which were not available for the current analysis, could help to achieve a better assessment of the dynamics of shape optimum shifts in future studies (Cooper, Thomas, Venditti, Meade, & Freckleton, 2016; Mahler, Weber, Wagner, & Ingram, 2017; Slater, Harmon, & Alfaro, 2012).

### 4.3 Trends in trait evolution according to likelihood scoring

As the OU1 model displayed the lowest SIC value regarding five out of nine scapular traits, we assume that the requirements on this skeletal element to resist and transmit forces are very similar between the fossorial and arboreal species. This is in agreement with Stalheim‐Smith (1984), who demonstrated on the basis of comparative physiological experiments on selected forelimb muscles of an arboreal and a fossorial sciuromorph rodent species that both have similar force‐generating potentials. In the few instances, in which differences were found, the arboreal species exceeded the fossorial one in its potential (Stalheim‐Smith, 1984). This also reflects the fact that the two fossorial lineages only evolved toward smaller trait optima in case they departed from the arboreal optimum. The only scapular traits in which both, Marmotini and Xerini, likely departed from the arboreal optimum concerned the two traits associated with the coracoid process. A longer mediolateral in‐lever of the muscles attaching to it allows for the generation of larger adduction forces, which may not be as important during digging as compared to climbing. Similarly, the strength of the articulation created by the coracoid process and the clavicula as determined by the size of the process appears to be of less importance. We think that this can be related to the relevance of mediolateral movements of the forelimb which are more likely to be required during climbing than digging.

Since the OU2_arb&Ma_ model never scored lowest and the OU2_arb&Xe_ model was most frequently suggested to be favorable among all traits according to the SIC (seven out of 16 cases), Marmotini rather than Xerini evolved away from the ancestral arboreal trait optimum. In five of these cases, it concerned femoral traits, indicating that differences in trait evolution between Marmotini and Xerini are more frequently found in the hind limb. As Marmotini displayed a smaller trait optimum value compared to Xerini and the arboreal group, this might hint at a relaxed functional constraint on the hind limb concerning its ability to resist and transmit forces. Perhaps this is related to differences in the utilization of the hind limb during digging between these two fossorial groups, leading to differing requirements on this skeletal element. Differences in how the hind limb is used (e.g., stabilization of the body during forelimb digging, hind limb digging, or pushing the body forwards when using the forelimbs to shovel away the soil) have been observed in other fossorial rodent taxa (Lehmann, 1963). Gambaryan (1974) argued that kicking the loose dirt is not a very strenuous behavior, but the forces which need to be generated during hind limb digging or resisted when the forelimbs are utilized might depend on the characteristics of the habitat. For example, it was shown that the soil hardness is reflected in the humeral morphology of different populations of the caviomorph rodent species *Ctenomys minutus* (Kubiak et al., 2018). Xerine species are found in arid habitats in Africa and Central Asia, whereas Marmotini is spread across the Holarctic region (Nowak, 1999; Thorington Jr. et al., 2012). The arid environment potentially contains harder soils, demanding larger forces during digging that in turn require a stronger stabilization and resistance to forces acting on the femur. However, personal qualitative observations on the smaller datasets used by Mielke et al. (2018) and Scheidt et al. (2019) indicated that all arboreal and fossorial species share overall a similar range of trait values in their trabecular and cross‐sectional robustness properties, respectively and hence, potentially, the same optima. However, their datasets need to be extended in order to enable an unbiased quantitative comparison with the traits used in our study. Furthermore, the interpretation of different environmental conditions is not reflected in the observed trend concerning scapular trait evolution. Here, it appears that Marmotini and Xerini share the same trait optimum (reflected in either the OU1 or the OU2_foss_ model having the lowest SIC score) in seven out of nine cases. Thus, although these findings provide an insight into the mosaic pattern of trait evolution in Sciuromorpha, they also open up new questions on the causes of the differences between the scapula and the femur demonstrated here and if these differences are reflected in traits concerning the inner bone morphology. The inclusion of such traits as well as more sophisticated data concerning the attaching muscles in future investigations might provide an even more differentiated insight into the trait evolution of these skeletal elements.

## 5 CONCLUSIONS

The independent acquisition of a fossorial lifestyle in mammals is a textbook example of homoplastic evolution of the postcranium (Hildebrand & Goslow, 1995). This is not supported by our study that utilized a rigorous phylogenetic comparative framework to study the most proximal limb bones of sciuromorph rodents. Our findings rather indicate a mosaic pattern of evolutionary processes depending on the skeletal element and trait under consideration. We provide evidence that the scapula tends to be constrained in more traits than the femur. This appears to be a result of shared functional demands between fossorial and arboreal species and not because of phylogenetic constraints. Contrary to this, the femur might be less constrained in its ability to resist and transmit forces. This suggests that its morphology depends on the requirements of the specific fossorial habitat, perhaps manifested in the biogeographical differences between Marmotini and Xerini. Comparative studies integrating habitat structure, behavior, performance, and biomechanics (as suggested by, e.g., Bock, 1980; Losos, 2011; Wainwright & Reilly, 1994) are necessary to test these conclusions and evaluate a more in‐depth form–function relationship in Sciuromorpha.

## CONFLICT OF INTEREST

The authors declare no conflict of interest.

## AUTHOR CONTRIBUTIONS

JW and JAN conceptualized the study. JW collected the data, conducted the data analysis, and drafted the manuscript. Both authors interpreted the results and revised previous versions of the manuscript.

## Data Availability

The data and R files associated with this manuscript are archived on Dryad (https://doi.org/10.5061/dryad.ng94td0).
