# Supplementary material for: Weighing homoplasy against alternative scenarios with the help of macroevolutionary modeling: A case study on limb bones of fossorial sciuromorph rodents
Source: Ecol Evol. 2019 Sep 9;9(19):11025–39. doi: 10.1002/ece3.5592 (PMC6802075; doi:10.1002/ece3.5592)
Supplement: Supplementary file 2 [file ECE3-9-11025-s002.docx]

**Supplementary material**

**Weighing homoplasy against alternative scenarios with the help of macroevolutionary modeling: A case study on limb bones of fossorial sciuromorph rodents**

**Authors and Affiliations**

Jan Wölfer^1,2,*^, John A. Nyakatura^1,2^

^1^AG Morphologie und Formengeschichte, Institut für Biologie, Humboldt-Universität zu Berlin, Philippstraße 13, 10115 Berlin, Germany

^2^*Bild Wissen Gestaltung.* Ein Interdisziplinäres Labor, Humboldt-Universität zu Berlin, Sophienstraße 22a, 10178 Berlin, Germany

**Corresponding author**

*Corresponding author: jan.woelfer@gmx.de

**Materials and Methods**

*S1: Mass comparison among Marmotini, Xerini, and arboreal species*

We pruned the datasets and the raw phylogeny so that they matched in species composition, because these pruned datasets were later used during evolutionary model comparison. Body mass information was taken from the literature (Tables S1 & S2). The natural log transformed mass values and not the actual mass values were tested as the former were used in all regression analyses to obtain residuals (see below) and thus, determine the differences among the three groups (arboreal species, Marmotini, and Xerini). The *aov* function was applied, followed by the *TukeyHSD* function including a 95% confidence level to correct the p-values of the pairwise comparisons of groups post hoc. The results (Tables S3 & S4) were very similar for the species included in the scapular and the femoral datasets, most likely because the sampled species were also similar. The difference between the two fossorial taxa was not significant (p = 0.999 for the scapular dataset and p = 0.977 for the femoral dataset). However, the mass range between each fossorial group and the arboreal group differed significantly (p = 5e-07 and p = 1e-07 for Marmotini and p = 0.041 and p = 0.042 for Xerini [scapula and femur, respectively]).

*S2: Simulation study to assess sampling bias*

For the simulation study, we decided to select three species along the sciuromorph mass range that are well represented in museum collections in order to assess the intraspecific variability for the scapular and femoral datasets, respectively. With increasing body mass, these were *Tamias striatus* (19 individuals including the specimen of the original dataset), *Sciurus carolinensis* (20), and *Marmota monax* (20) for the scapular dataset, and *Tamiasciurus hudsonicus* (18), *Sciurus carolinensis* (22), and *Marmota monax* (23) concerning the femoral dataset (Table S7). We collected both sexes equally well and as many subspecies as available. Each of the four species displays a broad geographic distribution that is above average compared among all sciuromorph species (Nowak, 1999; Thorington Jr., Koprowski, Steele, & Whatton, 2012), ensuring the coverage of a large intraspecific variation. The univariate traits were acquired as outlined in Wölfer, Arnold, and Nyakatura (2019) and Wölfer, Amson, et al. (2019). For each trait of each skeletal element, the following procedure was applied. The intraspecific standard deviation of the trait (not natural log transformed, yet) was computed for all three species. As the standard deviation appeared to depend on the size of the species (see Table S8), we used the *lm* function to regress the natural log of the standard deviations of the three species on their natural log transformed body mass. The regression equations were used to compute the expected standard deviations for all species included in the unpruned datasets (186 for the scapula and 177 for the femur; see Tables S1 & S2): intercept + slope * body mass. Then, the expected standard deviations were retransformed from natural log-transformed values using the *exp* function. We assumed the specimens’ trait values of the original dataset (the mean for the femoral traits of the three specimens of *Aplodontia rufa* being the only exception) to represent the mean species’ values, as the true values were not known. Given this and the estimated standard deviations for all species, a normal distribution of 1000 trait values was generated for each species of the original unpruned dataset, representing a hypothetical population (in statistical terms). The next step was repeated 1000 times. A sample was drawn with one specimen per species and the whole analysis was redone, starting with the natural log transformation of the trait values and the regression on mass, followed by the extraction of residuals and the pruning of the dataset according to the phylogeny, and completed with the likelihood estimation of the six evolutionary models via SIC. Based on all 1000 sampling results, we computed the frequency of the each model being the most likely one. All those traits for which the most frequent model did not match the most likely model estimated with the original dataset were dismissed from further analysis (Tables 1 & 2).


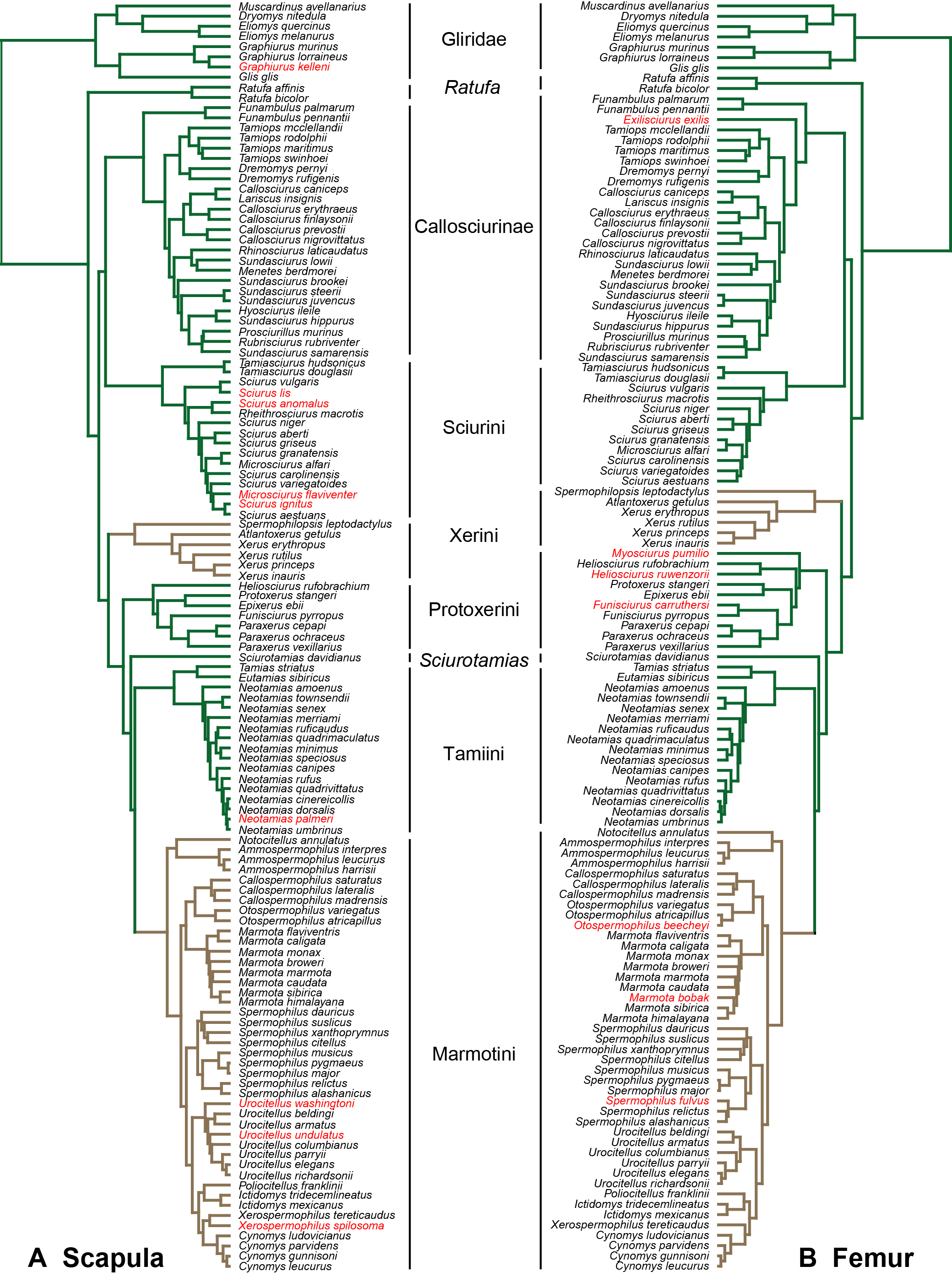


**Fig. A1. Phylogenies included in this study.** (A) Scapula, (B) Femur. Arboreal lineages in green, fossorial lineages in brown. Species in black included in both phylogenies, species in red exclusive to the respective phylogeny. The trees are part of a larger phylogeny assembled by Wölfer, Amson, et al. (2019) from phylogenetic information presented by Zelditch, Li, Tran, and Swiderski (2015) and the TimeTree database (Hedges, Marin, Suleski, Paymer, & Kumar, 2015). Names of Tamiini were updated with the suggested taxonomic revisions proposed by Patterson and Norris (2016).

**Table S1. List of specimens for the scapula.** Collection names are abbreviated as follows: Museum national d’Histoire naturelle (Paris, France), MNHN; Museum für Naturkunde (Berlin, Germany), ZMB; Centrum für Naturkunde (Hamburg, Germany), CeNaK; Staatliches Museum für Naturkunde (Stuttgart, Germany), SMNS; Phyletisches Museum (Jena, Germany), PMJ; Zoologische Staatssammlung (München, Germany), ZSM; Senckenberg Museum (Frankfurt, Germany), SMF; Smithsonian National Museum of Natural History (Washington, District of Columbia, USA), SNMNH; American Museum of National History (New York City, New York, USA), AMNH; University of Michigan Museum of Zoology (Ann Arbor, Michigan, USA), UMMZ; Michigan State University Museum (East Lansing, Michigan, USA), MSU; The Field Museum of Natural History (Chicago, Illinois, USA), FM. It is indicated in the column ‘PCM‘, whether a species was used in the phylogenetic comparative methods applied in this study. NA indicates missing information for the sex or body mass of a specimen. The column ‘Sex of body mass specimens’ indicates from which sex/sexes the body mass information was taken if not available for the specific sex of the sampled specimen. If the sex of the sampled specimen was unknown (NA), the mean body mass value of all information provided in the reference was computed. Body mass information was primarily taken from Hayssen (2008), otherwise as indicated by the column ‘Body mass reference’.

| **Species** | **collection** | **Catalog No.** | **PCM** | **Sex** | **Body mass [g]** | **Sex of body mass specimens** | **Body mass reference** |
| --- | --- | --- | --- | --- | --- | --- | --- |
| *Aeromys tephromelas* | SNMNH | 196743 | no | f | 1254 |  |  |
| *Aeromys thomasi* | FM | 90437 | no | f | 1117 |  |  |
| *Ammospermophilus harrisii* | SNMNH | 532504 | yes | f | 98 |  |  |
| *Ammospermophilus interpres* | MSU | MR.17752 | yes | f | 93 |  |  |
| *Ammospermophilus leucurus* | SNMNH | 578030 | yes | m | 95 |  |  |
| *Aplodontia rufa* | SNMNH | 192618 | no | m | 806 | mean of males and females | (Carraway & Verts, 1993) |
| *Atlantoxerus getulus* | SNMNH | 476806 | yes | f | 217 |  |  |
| *Callosciurus adamsi* | SNMNH | 396656 | no | f | 150 |  |  |
| *Callosciurus baluensis* | SNMNH | 292609 | no | f | 371 | unknown |  |
| *Callosciurus caniceps* | FM | 66164 | yes | m | 316 |  |  |
| *Callosciurus erythraeus* | SNMNH | 320819 | yes | m | 359 |  |  |
| *Callosciurus finlaysonii* | SNMNH | 584418 | yes | m | 278 | unknown |  |
| *Callosciurus nigrovittatus* | SNMNH | 294035 | yes | f | 239 |  |  |
| *Callosciurus notatus* | SNMNH | 548412 | no | f | 228 |  |  |
| *Callosciurus prevostii* | ZMB | Mam_88873 | yes | f | 362 |  |  |
| *Callosciurus pygerythrus* | FM | 104201 | no | m | 252 | unknown |  |
| *Callospermophilus lateralis* | UMMZ | 56214 | yes | f | 160 |  |  |
| *Callospermophilus madrensis* | MSU | 33277 | yes | f | 152 |  |  |
| *Callospermophilus saturatus* | UMMZ | 95781 | yes | m | 237 |  |  |
| *Cynomys gunnisoni* | SNMNH | 349306 | yes | m | 814 |  |  |
| *Cynomys leucurus* | SNMNH | A 11624 | yes | f | 924 |  |  |
| *Cynomys ludovicianus* | SNMNH | 511208 | yes | m | 849 |  |  |
| *Cynomys parvidens* | SNMNH | 397147 | yes | f | 516 |  |  |
| *Dremomys pernyi* | SNMNH | 574326 | yes | f | 151 |  |  |
| *Dremomys rufigenis* | SNMNH | 584419 | yes | f | 198 |  |  |
| *Dryomys nitedula* | SMF | 46395 | yes | m | 33 | mean of males | (Angermann, 1963) |
| *Eliomys melanurus* | SMF | 95257 | yes | f | 45 | only one female | (Shehab, Asaad, & Mamkhair, 2009) |
| *Eliomys quercinus* | PMJ | Mam92 | yes | NA | 78 | mean of males and females | (Bertolino, Cordero, & Currado, 2003) |
| *Eoglaucomys fimbriatus* | SNMNH | 353243 | no | f | 560 |  |  |
| *Epixerus ebii* | FM | 62225 | yes | m | 652 |  |  |
| *Funambulus palmarum* | SNMNH | 277234 | yes | NA | 108 | mean of male and female |  |
| *Funambulus pennantii* | SNMNH | 328003 | yes | f | 103 |  |  |
| *Funisciurus anerythreus* | FM | 149420 | no | f | 218 | unknown |  |
| *Funisciurus congicus* | CeNaK | S-1863 | no | f | 111 | unknown |  |
| *Funisciurus isabella* | SNMNH | 539408 | no | f | 107 | unknown |  |
| *Funisciurus lemniscatus* | UMMZ | 75275 | no | f | 141 | unknown |  |
| *Funisciurus leucogenys* | FM | 42658 | no | m | 271 |  |  |
| *Funisciurus pyrropus* | SNMNH | 539425 | yes | m | 225 |  |  |
| *Glaucomys sabrinus* | UMMZ | 54711 | no | f | 141 |  |  |
| *Glaucomys volans* | UMMZ | 178202 | no | f | 58 |  |  |
| *Glis glis* | SNMNH | 341581 | yes | m | 118 | mean of adult males | (Fietz, Pflug, Schlund, & Tataruch, 2005) |
| *Graphiurus crassicaudatus* | MNHN | 1980-79 | no | m | 24 | mean of unknown | (Holden & Levine, 2009) |
| *Graphiurus kelleni* | MNHN | 1981-614 | yes | NA | 24 | mean of males and females | (Kastenmayer, Moak, Jeffress, & Elkins, 2010) |
| *Graphiurus lorraineus* | SMF | 88419 | yes | m | 17 | mean of unknown | (Holden & Levine, 2009) |
| *Graphiurus microtis* | SMF | 88256 | no | m | NA |  | no literature found |
| *Graphiurus murinus* | SMF | 86331 | yes | f | 25 | mean of unknown | (Webb & Skinner, 1996) |
| *Graphiurus nagtglasii* | MNHN | 1967-1511 | no | f | 63 | mean of females | http://projects.biodiversity.be/africanrodentia/taxonomy/show?family_id=10&genus_id=22&species_id=39#exports (March/10th/2017) |
| *Graphiurus surdus* | SMF | 87429 | no | m | 25 | mean of unknown | (Holden & Levine, 2009) |
| *Heliosciurus gambianus* | MNHN | 1961-1036 | no | f | 329 |  |  |
| *Heliosciurus mutabilis* | FM | 214879 | no | m | 333 |  |  |
| *Heliosciurus punctatus* | MNHN | 1970-1519 | no | m | 166 |  |  |
| *Heliosciurus rufobrachium* | SNMNH | 543109 | yes | f | 361 |  |  |
| *Heliosciurus undulatus* | FM | 151215 | no | m | 315 | unknown |  |
| *Hylopetes alboniger* | SMNS | 40249 | no | f | 255 | mean of male and unknown |  |
| *Hylopetes lepidus* | SMNS | 40252 | no | f | 43 |  |  |
| *Hylopetes nigripes* | FM | 63025 | no | f | 534 | unknown |  |
| *Hylopetes phayrei* | SMNS | 40340 | no | m | 142 | mean of female and unknown |  |
| *Hylopetes spadiceus* | MNHN | 1960-3678 | no | NA | 75 | mean of all |  |
| *Hyosciurus ileile* | AMNH | M-225462 | yes | f | 391 |  |  |
| *Ictidomys mexicanus* | UMMZ | 79344 | yes | m | 223 |  |  |
| *Ictidomys parvidens* | SNMNH | 588485 | no | f | 121 | mean of females | (Thorington Jr. et al., 2012) |
| *Ictidomys tridecemlineatus* | FM | 167033 | yes | f | 143 |  |  |
| *Iomys horsfieldi* | SNMNH | 292654 | no | f | 210 |  |  |
| *Lariscus hosei* | SNMNH | 449981 | no | m | 215 | female |  |
| *Lariscus insignis* | FM | 213418 | yes | m | 175 |  |  |
| *Marmota broweri* | SNMNH | 583154 | yes | m | 3630 |  |  |
| *Marmota caligata* | SNMNH | A 49815 | yes | f | 3515 |  |  |
| *Marmota caudata* | SNMNH | 173381 | yes | f | 5000 |  |  |
| *Marmota flaviventris* | SNMNH | 575170 | yes | m | 3909 |  |  |
| *Marmota himalayana* | SNMNH | 198637 | yes | m | 6000 | unknown |  |
| *Marmota marmota* | ZMB | Mam_44862 | yes | m | 4303 |  |  |
| *Marmota monax* | ummz | 166225 | yes | NA | 3036 | mean of all |  |
| *Marmota sibirica* | ZMB | 37902 | yes | f | 8000 | unknown |  |
| *Menetes berdmorei* | SNMNH | 583800 | yes | m | 176 |  |  |
| *Microsciurus alfari* | SNMNH | 305632 | yes | m | 83 |  |  |
| *Microsciurus flaviventer* | FM | 71119 | yes | m | 98 |  |  |
| *Microsciurus mimulus* | SNMNH | 338174 | no | m | 120 | mean of female and unknown |  |
| *Muscardinus avellanarius* | ZMB | 105826 | yes | NA | 17 | mean of unknown | (Csorba, 2003) |
| *Notocitellus annulatus* | UMMZ | 94630 | yes | f | 386 |  |  |
| *Otospermophilus atricapillus* | SNMNH | 528797 | yes | m | 505 |  |  |
| *Otospermophilus variegatus* | UMMZ | 79328 | yes | f | 673 |  |  |
| *Paraxerus cepapi* | SNMNH | 295201 | yes | f | 180 |  |  |
| *Paraxerus lucifer* | FM | 196631 | no | m | 680 |  |  |
| *Paraxerus ochraceus* | SNMNH | 590017 | yes | f | 138 |  |  |
| *Paraxerus poensis* | UMMZ | 75279 | no | f | 125 |  |  |
| *Paraxerus vexillarius* | FM | 197806 | yes | m | 243 |  |  |
| *Paraxerus vincenti* | FM | 183736 | no | f | 375 |  | (Kingdon, 2015) |
| *Petaurista alborufus* | SNMNH | 588833 | no | f | 1454 |  |  |
| *Petaurista elegans* | FM | 114372 | no | f | 760 |  |  |
| *Petaurista leucogenys* | SNMNH | A 20941 | no | NA | 1179 | unknown |  |
| *Petaurista magnificus* | FM | 114366 | no | f | 1800 |  |  |
| *Petaurista petaurista* | SNMNH | 588884 | no | m | 1264 |  |  |
| *Petinomys setosus* | SMNS | 40303 | no | m | 41 |  |  |
| *Petinomys vordermanni* | MNHN | 1979-346 | no | f | 36 | mean of male and female |  |
| *Poliocitellus franklinii* | SNMNH | 54153 | yes | f | 425 |  |  |
| *Prosciurillus abstrusus* | AMNH | M-225504 | no | f | 79 | unknown |  |
| *Prosciurillus murinus* | AMNH | M-225909 | yes | m | 73 | unknown |  |
| *Protoxerus stangeri* | UMMZ | 75274 | yes | f | 761 |  |  |
| *Pteromys momonga* | ZMB | 78564 | no | m | 152 | unknown |  |
| *Pteromys volans* | ZMB | 60468 | no | NA | 134 | mean of male and unknown |  |
| *Ratufa affinis* | SNMNH | 317202 | yes | m | 1064 |  |  |
| *Ratufa bicolor* | SNMNH | 573966 | yes | m | 1678 |  |  |
| *Ratufa indica* | SNMNH | 322077 | no | NA | 1441 | mean of all |  |
| *Ratufa macroura* | ZSM | 1905-1082 | no | m | 1610 |  |  |
| *Rheithrosciurus macrotis* | SNMNH | 197261 | yes | f | 1308 |  |  |
| *Rhinosciurus laticaudatus* | FM | 98526 | yes | m | 242 |  |  |
| *Rubrisciurus rubriventer* | AMNH | M-225490 | yes | f | 673 |  |  |
| *Sciurotamias davidianus* | SNMNH | 258511 | yes | f | 260 | unknown |  |
| *Sciurus aberti* | SNMNH | 448233 | yes | m | 594 |  |  |
| *Sciurus aestuans* | SNMNH | 548445 | yes | f | 200 |  |  |
| *Sciurus alleni* | UMMZ | 61447 | no | m | 447 |  |  |
| *Sciurus anomalus* | UMMZ | 101188 | yes | m | 345 |  |  |
| *Sciurus arizonensis* | UMMZ | 66355 | no | m | 736 |  |  |
| *Sciurus aureogaster* | AMNH | M-190229 | no | f | 505 |  |  |
| *Sciurus carolinensis* | SNMNH | 548056 | yes | f | 513 |  |  |
| *Sciurus colliaei* | UMMZ | 99913 | no | m | 335 |  |  |
| *Sciurus deppei* | SNMNH | 244937 | no | f | 287 |  |  |
| *Sciurus gilvigularis* | SNMNH | 549526 | no | m | 158 | mean of unknown | (Thorington Jr. et al., 2012) |
| *Sciurus granatensis* | UMMZ | 112048 | yes | m | 300 |  |  |
| *Sciurus griseus* | SNMNH | A 21513 | yes | NA | 770 | mean of female and unknown |  |
| *Sciurus ignitus* | AMNH | M-239991 | yes | f | 222 | unknown |  |
| *Sciurus igniventris* | FM | 47600 | no | f | 700 | unknown |  |
| *Sciurus ingrami* | FM | 94370 | no | m | NA |  | no literature found |
| *Sciurus lis* | SNMNH | 582434 | yes | m | 176 |  |  |
| *Sciurus nayaritensis* | UMMZ | 99958 | no | f | 756 |  |  |
| *Sciurus niger* | SNMNH | 583325 | yes | f | 764 |  |  |
| *Sciurus oculatus* | UMMZ | 114038 | no | f | 582 |  |  |
| *Sciurus pucheranii* | FM | 70041 | no | f | 100 | unknown |  |
| *Sciurus pyrrhinus* | AMNH | M-60507 | no | m | NA |  | no literature found |
| *Sciurus spadiceus* | FM | 122956 | no | f | 513 |  |  |
| *Sciurus variegatoides* | SNMNH | 398850 | yes | m | 537 |  |  |
| *Sciurus vulgaris* | UMMZ | 97337 | yes | NA | 379 | mean of all |  |
| *Sciurus yucatanensis* | UMMZ | 64050 | no | f | 302 |  |  |
| *Spermophilopsis leptodactylus* | SNMNH | 545230 | yes | f | 548 |  |  |
| *Spermophilus alashanicus* | ZMB | Mam_62232 | yes | f | 208 |  | (Smith et al., 2010) |
| *Spermophilus citellus* | UMMZ | 123540 | yes | f | 202 |  |  |
| *Spermophilus dauricus* | UMMZ | 123543 | yes | f | 224 | unknown |  |
| *Spermophilus major* | UMMZ | 92750 | yes | m | 535 | mean of unknown | (Thorington Jr. et al., 2012) |
| *Spermophilus musicus* | UMMZ | 123549 | yes | NA | 310 | mean of males and females | (Kryštufek & Vohralík, 2012) |
| *Spermophilus pygmaeus* | UMMZ | 123552 | yes | f | 235 | unknown |  |
| *Spermophilus relictus* | UMMZ | 123554 | yes | m | 260 | mean of males | (Kryštufek & Vohralík, 2012) |
| *Spermophilus suslicus* | UMMZ | 123555 | yes | NA | 218 | mean of male and female |  |
| *Spermophilus xanthoprymnus* | UMMZ | 123559 | yes | m | 311 | unknown |  |
| *Sundasciurus brookei* | ZMB | Mam_88864 | yes | m | 124 |  |  |
| *Sundasciurus hippurus* | SNMNH | 449982 | yes | m | 430 |  |  |
| *Sundasciurus hoogstraali* | SNMNH | 477858 | no | m | NA |  | no literature found |
| *Sundasciurus juvencus* | FM | 63058 | yes | f | 245 |  |  |
| *Sundasciurus lowii* | SNMNH | 396659 | yes | f | 76 |  |  |
| *Sundasciurus samarensis* | SNMNH | 458743 | yes | m | 243 |  |  |
| *Sundasciurus steerii* | FM | 63109 | yes | m | 257 |  |  |
| *Tamias amoenus* | UMMZ | 59680 | yes | f | 51 |  |  |
| *Tamias canipes* | UMMZ | 79067 | yes | m | 70 | unknown |  |
| *Tamias cinereicollis* | UMMZ | 56245 | yes | f | 72 |  |  |
| *Tamias dorsalis* | UMMZ | 64007 | yes | m | 65 |  |  |
| *Tamias merriami* | UMMZ | 173663 | yes | f | 70 | mean of male and unknown |  |
| *Tamias minimus* | UMMZ | 167071 | yes | m | 44 |  |  |
| *Tamias palmeri* | FM | 140578 | yes | f | 55 |  |  |
| *Tamias quadrimaculatus* | SNMNH | 398282 | yes | m | 78 |  |  |
| *Tamias quadrivittatus* | AMNH | M-137897 | yes | f | 63 |  |  |
| *Tamias ruficaudus* | UMMZ | 162564 | yes | f | 63 |  |  |
| *Tamias rufus* | SNMNH | 564127 | yes | f | 58 |  |  |
| *Tamias senex* | UMMZ | 54332 | yes | f | 94 |  |  |
| *Tamias sibiricus* | CeNak | S-7138 | yes | f | 96 |  |  |
| *Tamias speciosus* | UMMZ | 177079 | yes | f | 63 |  |  |
| *Tamias striatus* | ZMB | 88869 | yes | NA | 97 | mean of male and female |  |
| *Tamias townsendii* | UMMZ | 54331 | yes | m | 70 |  |  |
| *Tamias umbrinus* | UMMZ | 61979 | yes | m | 56 |  |  |
| *Tamiasciurus douglasii* | SNMNH | 549145 | yes | m | 207 |  |  |
| *Tamiasciurus hudsonicus* | UMMZ | 67345 | yes | f | 213 |  |  |
| *Tamiops maritimus* | SMF | 86733 | yes | m | 55 |  |  |
| *Tamiops mcclellandii* | SMF | 53438 | yes | m | 50 |  |  |
| *Tamiops rodolphii* | MNHN | 1990-535 | yes | m | 56 | unknown |  |
| *Tamiops swinhoei* | SNMNH | 574325 | yes | f | 88 |  |  |
| *Urocitellus armatus* | SNMNH | 484947 | yes | m | 395 |  |  |
| *Urocitellus beldingi* | FM | 219749 | yes | f | 265 |  |  |
| *Urocitellus canus* | UMMZ | 54669 | no | f | 154 | unknown |  |
| *Urocitellus columbianus* | SNMNH | 398301 | yes | m | 490 |  |  |
| *Urocitellus elegans* | SNMNH | 552418 | yes | m | 330 |  |  |
| *Urocitellus mollis* | SNMNH | 484968 | no | f | 115 |  |  |
| *Urocitellus parryii* | SNMNH | A 34844 | yes | f | 524 |  |  |
| *Urocitellus richardsonii* | SNMNH | 398240 | yes | f | 273 |  |  |
| *Urocitellus townsendii* | SNMNH | 89977 | no | m | 250 |  |  |
| *Urocitellus undulatus* | ZMB | 62186 | yes | f | 718 |  |  |
| *Urocitellus washingtoni* | UMMZ | 54641 | yes | f | 187 |  |  |
| *Xerospermophilus spilosoma* | FM | 125175 | yes | m | 128 |  |  |
| *Xerospermophilus terticaudus* | UMMZ | 63860 | yes | m | 124 |  |  |
| *Xerus erythropus* | AMNH | M-51351 | yes | f | 742 |  |  |
| *Xerus inauris* | SNMNH | 295222 | yes | f | 580 |  |  |
| *Xerus princeps* | CeNaK | 1441 | yes | f | 665 | unknown |  |
| *Xerus rutilus* | AMNH | M-187452 | yes | m | 307 |  |  |

**Table S2: List of specimens for the femur.** See Table S1 for abbreviations of collection names. It is indicated in the column ‘PCM‘, whether a species was used in the phylogenetic comparative methods applied in this study. NA indicates missing information for the sex or body mass of a specimen. The column ‘Sex of body mass specimens’ indicates from which sex/sexes the body mass information was taken if not available for the specific sex of the sampled specimen. If the sex of the sampled specimen was unknown (NA), the mean body mass value of all information provided in the reference was computed. Body mass information was primarily taken from Hayssen (2008), otherwise as indicated by the column ‘Body mass reference’.

| Species | Collection | Catalog No. | PCM | Sex | Body mass [g] | Sex of body mass specimens | Body mass reference |
| --- | --- | --- | --- | --- | --- | --- | --- |
| *Aeromys tephromelas* | SNMNH | 196743 | no | f | 1254 |  |  |
| *Aeromys thomasi* | FM | 90437 | no | f | 1117 |  |  |
| *Ammospermophilus harrisii* | UMMZ | 61736 | yes | m | 116 |  |  |
| *Ammospermophilus interpres* | AMNH | M-131944 | yes | f | 93 |  |  |
| *Ammospermophilus leucurus* | UMMZ | 80988 | yes | m | 95 |  |  |
| *Aplodontia rufa* | SNMNH | 192618 | no | m | 806 | mean of males and females | (Carraway & Verts, 1993) |
| *Aplodontia rufa* | ZMB | Mam_20983 | no | NA | 806 | mean of males and females | (Carraway & Verts, 1993) |
| *Aplodontia rufa* | ZMB | Mam_20987 | no | m | 806 | mean of males and females | (Carraway & Verts, 1993) |
| *Atlantoxerus getulus* | SMF | 48688 | yes | f | 217 |  |  |
| *Callosciurus adamsi* | SNMNH | 396656 | no | f | 150 |  |  |
| *Callosciurus baluensis* | SNMNH | 292609 | no | f | 371 | unknown |  |
| *Callosciurus caniceps* | FM | 66164 | yes | m | 316 |  |  |
| *Callosciurus erythraeus* | SNMNH | 255936 | yes | m | 359 |  |  |
| *Callosciurus finlaysonii* | SNMNH | 584417 | yes | f | 278 | unknown |  |
| *Callosciurus nigrovittatus* | MNHN | ZM-MO-1977-570 | yes | f | 239 |  |  |
| *Callosciurus notatus* | SNMNH | 548412 | no | f | 228 |  |  |
| *Callosciurus prevostii* | ZMB | Mam_88873 | yes | f | 362 |  |  |
| *Callosciurus pygerythrus* | FM | 104198 | no | f | 252 | unknown |  |
| *Callospermophilus lateralis* | UMMZ | 56217 | yes | f | 160 |  |  |
| *Callospermophilus madrensis* | MSU | MR.33277 | yes | f | 152 |  |  |
| *Callospermophilus saturatus* | UMMZ | 54615 | yes | m | 237 |  |  |
| *Cynomys gunnisoni* | SNMNH | 533015 | yes | m | 814 |  |  |
| *Cynomys leucurus* | SNMNH | A 11624 | yes | f | 924 |  |  |
| *Cynomys ludovicianus* | SNMNH | 511208 | yes | m | 849 |  |  |
| *Cynomys parvidens* | FM | 106739 | yes | m | 636 |  |  |
| *Dremomys pernyi* | SNMNH | 574326 | yes | f | 151 |  |  |
| *Dremomys rufigenis* | SNMNH | 584419 | yes | f | 198 |  |  |
| *Dryomys nitedula* | SMF | 44514 | yes | f | 31 | mean of females | (Angermann, 1963) |
| *Eliomys melanurus* | SMF | 95257 | yes | f | 45 | only one female | (Shehab et al., 2009) |
| *Eliomys quercinus* | UMMZ | 99272 | yes | f | 75 | mean of females | (Bertolino et al., 2003) |
| *Eoglaucomys fimbriatus* | SNMNH | 353243 | no | f | 560 |  |  |
| *Epixerus ebii* | SNMNH | 539397 | yes | f | 388 |  |  |
| *Exilisciurus exilis* | FM | 76874 | yes | m | 17 |  |  |
| *Funambulus palmarum* | CeNaK | S-4888 | yes | m | 118 | mean of female and male |  |
| *Funambulus pennantii* | CeNaK | S-4882 | yes | m | 95 | mean of female and male |  |
| *Funisciurus anerythreus* | ZMB | Mam_18337 | no | f | 218 | unknown |  |
| *Funisciurus carruthersi* | FM | 137640 | yes | f | 268 | unknown |  |
| *Funisciurus congicus* | CeNaK | S-1863 | no | f | 111 | unknown |  |
| *Funisciurus isabella* | SNMNH | 539407 | no | f | 107 | unknown |  |
| *Funisciurus lemniscatus* | SNMNH | 539410 | no | m | 141 | unknown |  |
| *Funisciurus leucogenys* | MNHN | ZM-MO-1961-306 | no | f | 252 |  |  |
| *Funisciurus pyrropus* | SNMNH | 539425 | yes | m | 225 |  |  |
| *Glaucomys sabrinus* | UMMZ | 54711 | no | f | 141 |  |  |
| *Glaucomys volans* | UMMZ | 178202 | no | f | 58 |  |  |
| *Glis glis* | SNMNH | 341581 | yes | m | 118 | mean of adult males | (Fietz et al., 2005) |
| *Graphiurus lorraineus* | MNHN | ZM-MO-1980-87 | yes | m | 17 | mean of unknow sex | (Holden & Levine, 2009) |
| *Graphiurus murinus* | SNMNH | 548527 | yes | m | 25 | mean of unknown sex | (Webb & Skinner, 1996) |
| *Graphiurus nagtglasii* | MNHN | ZM-MO-1967-1418 | no | f | 63 | mean of females | http://projects.biodiversity.be/africanrodentia/taxonomy/show?family_id=10&genus_id=22&species_id=39#exports (March/10th/2017) |
| *Heliosciurus gambianus* | MNHN | ZM-MO-1961-1036 | no | f | 329 |  |  |
| *Heliosciurus mutabilis* | FM | 212091 | no | f | 383 |  |  |
| *Heliosciurus punctatus* | MNHN | ZM-MO-1970-519 | no | m | 166 |  |  |
| *Heliosciurus rufobrachium* | SNMNH | 543109 | yes | f | 361 |  |  |
| *Heliosciurus ruwenzorii* | FM | 149004 | yes | m | 305 |  |  |
| *Hylopetes alboniger* | SMNS | 40249 | no | f | 255 | mean of male and unknown |  |
| *Hylopetes lepidus* | SMNS | 40252 | no | f | 43 |  |  |
| *Hylopetes nigripes* | FM | 63025 | no | f | 534 | unknown |  |
| *Hylopetes phayrei* | SMNS | 40340 | no | m | 142 | mean of female and unknown |  |
| *Hylopetes spadiceus* | MNHN | ZM-MO-1979-372 | no | f | 78 |  |  |
| *Hyosciurus ileile* | AMNH | M-225463 | yes | m | 398 |  |  |
| *Ictidomys mexicanus* | AMNH | M-203961 | yes | f | 167 |  |  |
| *Ictidomys tridecemlineatus* | UMMZ | 162762 | yes | f | 143 |  |  |
| *Lariscus hosei* | SNMNH | 449981 | no | m | 215 | female |  |
| *Lariscus insignis* | ANMNH | M-106417 | yes | m | 175 |  |  |
| *Marmota bobak* | AMNH | M-45701 | yes | f | 3875 |  |  |
| *Marmota broweri* | SNMNH | 583154 | yes | m | 3630 |  |  |
| *Marmota caligata* | SNMNH | A 49815 | yes | f | 3515 |  |  |
| *Marmota caudata* | SNMNH | 173381 | yes | f | 5000 |  |  |
| *Marmota flaviventris* | SNMNH | 575170 | yes | m | 3909 |  |  |
| *Marmota himalayana* | SNMNH | 198637 | yes | m | 6000 | unknown |  |
| *Marmota marmota* | ZMB | Mam_44862 | yes | m | 4303 |  |  |
| *Marmota monax* | ZMB | Mam_12055 | yes | NA | 3036 | mean of all |  |
| *Marmota sibirica* | ZMB | Mam_37956 | yes | NA | 8000 | unknown |  |
| *Menetes berdmorei* | SMF | 53433 | yes | f | 172 |  |  |
| *Microsciurus alfari* | SNMNH | 305632 | yes | m | 83 |  |  |
| *Microsciurus mimulus* | SNMNH | 338174 | no | m | 120 | mean of female and unknown |  |
| *Muscardinus avellanarius* | ZMB | 105826 | yes | NA | 17 | mean of unknown | (Csorba, 2003) |
| *Myosciurus pumilio* | SNMNH | 539436 | yes | m | 16 | unknown |  |
| *Notocitellus annulatus* | SNMNH | 88138 | yes | f | 386 |  |  |
| *Otospermophilus atricapillus* | SNMNH | 528797 | yes | m | 505 |  |  |
| *Otospermophilus beecheyi* | ZMB | Mam_16619 | yes | f | 509 |  |  |
| *Otospermophilus variegatus* | FM | 47176 | yes | f | 673 |  |  |
| *Paraxerus cepapi* | SNMNH | 295201 | yes | m | 186 |  |  |
| *Paraxerus lucifer* | FM | 196631 | no | m | 680 |  |  |
| *Paraxerus ochraceus* | SNMNH | 590017 | yes | m | 125 |  |  |
| *Paraxerus poensis* | SNMNH | 539389 | no | m | 115 |  |  |
| *Paraxerus vexillarius* | FM | 197806 | yes | m | 243 |  |  |
| *Paraxerus vincenti* | FM | 183736 | no | f | 375 |  | (Kingdon, 2015) |
| *Petaurista alborufus* | SNMNH | 588833 | no | f | 1454 |  |  |
| *Petaurista elegans* | FM | 114372 | no | f | 760 |  |  |
| *Petaurista leucogenys* | SNMNH | A 20941 | no | NA | 1179 | unknown |  |
| *Petaurista magnificus* | FM | 114364 | no | m | 1800 | female |  |
| *Petaurista petaurista* | SNMNH | 588884 | no | m | 1264 |  |  |
| *Petaurista philippensis* | SNMNH | 314973 | no | m | 2268 | unknown |  |
| *Petinomys hageni* | PMJ | Mam3362 | no | f | 346 |  |  |
| *Petinomys setosus* | SMNS | 40303 | no | m | 41 |  |  |
| *Petinomys vordermanni* | MNHN | ZM-MO-1979-346 | no | f | 36 | mean of female and male |  |
| *Poliocitellus franklinii* | FM | 56859 | yes | f | 425 |  |  |
| *Prosciurillus abstrusus* | AMNH | M-225504 | no | f | 79 | unknown |  |
| *Prosciurillus murinus* | AMNH | M-225492 | yes | f | 73 | unknown |  |
| *Protoxerus aubinii* | AMNH | M-239341 | no | m | 415 |  |  |
| *Protoxerus stangeri* | SNMNH | 237333 | yes | m | 538 |  |  |
| *Pteromys momonga* | ZMB | Mam_78564 | no | m | 152 | unknown |  |
| *Pteromys volans* | ZMB | Mam_60468 | no | NA | 134 | mean of male and unknown |  |
| *Ratufa affinis* | SNMNH | 317202 | yes | m | 1064 |  |  |
| *Ratufa bicolor* | SNMNH | 573966 | yes | m | 1678 |  |  |
| *Ratufa indica* | SNMNH | 322077 | no | NA | 1441 | mean of all |  |
| *Ratufa macroura* | ZSM | 1905-1082 | no | m | 1610 |  |  |
| *Rheithrosciurus macrotis* | SNMNH | 197261 | yes | f | 1308 |  |  |
| *Rhinosciurus laticaudatus* | FM | 98525 | yes | f | 233 |  |  |
| *Rubrisciurus rubriventer* | AMNH | M-226053 | yes | m | 703 |  |  |
| *Sciurotamias davidianus* | SNMNH | 258511 | yes | f | 260 | unknown |  |
| *Sciurus aberti* | FM | 122727 | yes | NA | 605 | mean of all |  |
| *Sciurus aestuans* | AMNH | M-267565 | yes | f | 200 |  |  |
| *Sciurus alleni* | UMMZ | 61447 | no | m | 447 |  |  |
| *Sciurus arizonensis* | UMMZ | 66355 | no | m | 736 |  |  |
| *Sciurus aureogaster* | UMMZ | 109354 | no | m | 497 |  |  |
| *Sciurus carolinensis* | SNMNH | 548056 | yes | f | 513 |  |  |
| *Sciurus colliaei* | UMMZ | 99913 | no | m | 335 |  |  |
| *Sciurus deppei* | SNMNH | 244937 | no | f | 287 |  |  |
| *Sciurus granatensis* | SNMNH | 578378 | yes | f | 312 |  |  |
| *Sciurus griseus* | SNMNH | A 21513 | yes | NA | 770 | mean of female and unknown |  |
| *Sciurus igniventris* | FM | 47600 | no | f | 700 | unknown |  |
| *Sciurus ingrami* | FM | 94370 | no | m | NA |  | no literature found |
| *Sciurus nayaritensis* | UMMZ | 99958 | no | f | 756 |  |  |
| *Sciurus niger* | SNMNH | 583325 | yes | f | 764 |  |  |
| *Sciurus oculatus* | UMMZ | 114038 | no | f | 582 |  |  |
| *Sciurus pyrrhinus* | AMNH | M-60507 | no | m | NA |  | no literature found |
| *Sciurus spadiceus* | AMNH | M-211692 | no | f | 513 |  |  |
| *Sciurus variegatoides* | SNMNH | 398850 | yes | m | 537 |  |  |
| *Sciurus vulgaris* | SMNS | 46104 | yes | f | 371 |  |  |
| *Sciurus yucatanensis* | SNMNH | 244943 | no | f | 302 |  |  |
| *Spermophilopsis leptodactylus* | SNMNH | 545230 | yes | f | 548 |  |  |
| *Spermophilus alashanicus* | ZMB | Mam_62174 | yes | f | 208 |  | (Smith et al., 2010) |
| *Spermophilus citellus* | UMMZ | 123540 | yes | f | 202 |  |  |
| *Spermophilus dauricus* | UMMZ | 123543 | yes | f | 224 | unknown |  |
| *Spermophilus fulvus* | UMMZ | 123544 | yes | m | 290 |  |  |
| *Spermophilus major* | UMMZ | 92750 | yes | m | 535 | mean of unknown | (Thorington Jr. et al., 2012) |
| *Spermophilus musicus* | UMMZ | 123549 | yes | NA | 310 | mean of males and females | (Kryštufek & Vohralík, 2012) |
| *Spermophilus pygmaeus* | UMMZ | 123552 | yes | f | 235 | unknown |  |
| *Spermophilus relictus* | UMMZ | 123554 | yes | m | 260 |  | (Kryštufek & Vohralík, 2012) |
| *Spermophilus suslicus* | UMMZ | 123555 | yes | NA | 218 | mean of female and male |  |
| *Spermophilus xanthoprymnus* | UMMZ | 123559 | yes | m | 311 | unknown |  |
| *Sundasciurus brookei* | ZMB | Mam_88864 | yes | m | 124 |  |  |
| *Sundasciurus hippurus* | SNMNH | 449982 | yes | m | 430 |  |  |
| *Sundasciurus hoogstraali* | SNMNH | 477858 | no | m | NA |  | no literature found |
| *Sundasciurus juvencus* | FM | 63036 | yes | f | 245 |  |  |
| *Sundasciurus lowii* | SNMNH | 396659 | yes | f | 76 |  |  |
| *Sundasciurus samarensis* | SNMNH | 458740 | yes | m | 243 |  |  |
| *Sundasciurus steerii* | SNMNH | 477968 | yes | m | 257 |  |  |
| *Tamias amoenus* | UMMZ | 59680 | yes | f | 51 |  |  |
| *Tamias canipes* | UMMZ | 79067 | yes | m | 70 | unknown |  |
| *Tamias cinereicollis* | UMMZ | 56245 | yes | f | 72 |  |  |
| *Tamias dorsalis* | UMMZ | 64007 | yes | m | 65 |  |  |
| *Tamias merriami* | UMMZ | 173663 | yes | f | 70 | mean of male and unknown |  |
| *Tamias minimus* | UMMZ | 167071 | yes | m | 44 |  |  |
| *Tamias quadrimaculatus* | SNMNH | 398282 | yes | m | 78 |  |  |
| *Tamias quadrivittatus* | AMNH | M-137897 | yes | f | 63 |  |  |
| *Tamias ruficaudus* | UMMZ | 162564 | yes | f | 63 |  |  |
| *Tamias rufus* | SNMNH | 564127 | yes | f | 58 |  |  |
| *Tamias senex* | UMMZ | 54332 | yes | f | 94 |  |  |
| *Tamias sibiricus* | SMF | 56497 | yes | f | 96 |  |  |
| *Tamias speciosus* | SNMNH | 398283 | yes | f | 63 |  |  |
| *Tamias striatus* | UMMZ | 67521 | yes | m | 101 |  |  |
| *Tamias townsendii* | AMNH | M-121126 | yes | f | 76 |  |  |
| *Tamias umbrinus* | UMMZ | 61979 | yes | m | 56 |  |  |
| *Tamiasciurus douglasii* | UMMZ | 176155 | yes | m | 207 |  |  |
| *Tamiasciurus hudsonicus* | UMMZ | 112423 | yes | m | 194 |  |  |
| *Tamiops maritimus* | SMF | 86733 | yes | m | 55 |  |  |
| *Tamiops mcclellandii* | SMF | 53442 | yes | m | 50 |  |  |
| *Tamiops rodolphii* | MNHN | ZM-MO-1982-24 | yes | m | 56 | unknown |  |
| *Tamiops swinhoei* | SNMNH | 256111 | yes | f | 88 |  |  |
| *Urocitellus armatus* | SNMNH | 484947 | yes | m | 395 |  |  |
| *Urocitellus beldingi* | SNMNH | 271149 | yes | m | 229 |  |  |
| *Urocitellus columbianus* | SNMNH | 398301 | yes | m | 490 |  |  |
| *Urocitellus elegans* | SNMNH | 552418 | yes | m | 330 |  |  |
| *Urocitellus parryii* | SNMNH | A 34844 | yes | f | 524 |  |  |
| *Urocitellus richardsonii* | SNMNH | 398240 | yes | f | 273 |  |  |
| *Xerospermophilus terticaudus* | UMMZ | 63860 | yes | m | 124 |  |  |
| *Xerus erythropus* | AMNH | M-51351 | yes | f | 742 |  |  |
| *Xerus inauris* | SNMNH | 295222 | yes | f | 580 |  |  |
| *Xerus princeps* | CeNaK | S-1441 | yes | f | 665 | unknown |  |
| *Xerus rutilus* | AMNH | M-187452 | yes | m | 307 |  |  |

**Table S3. Tukey test for multiple comparison of mean body mass concerning the scapular dataset.** Arb = arboreal, Ma = Marmotini, Xe = Xerini. Diff = difference between groups, lwr = lower confidence limit, upr = upper confidence limit, P_adj_ = adjusted p value.

| Groups compared | diff | lwr | upr | P_adj_ |
| --- | --- | --- | --- | --- |
| Ma vs arb | 1.149 | 0.657 | 1.641 | **5e-07** |
| Xe vs arb | 1.129 | 0.035 | 2.223 | **0.041** |
| Xe vs ma | -0.020 | -1.144 | 1.104 | 0.999 |

**Table S4. Tukey test for multiple comparison of mean body mass concerning the femoral dataset.** Arb = arboreal, Ma = Marmotini, Xe = Xerini. Diff = difference between groups, lwr = lower confidence limit, upr = upper confidence limit, P_adj_ = adjusted p value.

| Groups compared | diff | lwr | upr | P_adj_ |
| --- | --- | --- | --- | --- |
| Ma vs arb | 1.259 | 0.752 | 1.767 | **1e-07** |
| Xe vs arb | 1.159 | 0.035 | 2.282 | **0.042** |
| Xe vs ma | -0.101 | -1.254 | 1.053 | 0.977 |

**Table S5. Estimated model parameters and their confidence intervals concerning the scapular dataset.** EL = effective length, CS = centroid size, ca = from caudal view, ve = from ventral view, I-L = in-lever, ml = mediolateral, I-L_ori_ = orientation of the in-lever in respect the axis of scapula EL, la = from lateral view. Asterisks indicates empirical parameter that always fell outside of its confidence interval.

| Model | Parameter | Scapula EL | Glenoid cavity CS_ca_ | Coracoid process CS | Coracoid process I-L_ml_ |
| --- | --- | --- | --- | --- | --- |
| BM1 | θ | -0.017 (-3.529, 3.497) | -0.014 (-3.219, 3.325) | 0.045 (-4.425, 4.666) | 0.041 (-3.724, 3.803) |
|  | σ* | 0.223 (9.917, 16.484) | 0.189 (8.475, 14.009) | 0.345 (15.519, 25.49) | 0.237 (10.621, 17.705) |
| OU1 | θ (all) | 0.009 (-0.008, 0.027) | -0.005 (-0.022, 0.012) | -0.015 (-0.058, 0.029) | 0.001 (-0.032, 0.033) |
|  | *t*_1/2_ | 0.249 (0.025, 1.827) | 0.254 (0.026, 1.755) | 9.698 (0.022, 1.86) | 7.238 (0.024, 1.811) |
|  | σ | 3.335 (0.445, 32.906) | 2.928 (0.41, 30.017) | 0.529 (2.755, 247.139) | 0.39 (1.557, 118.65) |
| OU2_foss_ | θ (arb) | 0.021 (-0.005, 0.046) | 0.008 (-0.015, 0.032) | 0.087 (0.035, 0.139) | 0.083 (0.045, 0.121) |
|  | θ (foss) | -0.001 (-0.025, 0.024) | -0.017 (-0.04, 0.006) | -0.242 (-0.293, -0.19) | -0.148 (-0.183, -0.113) |
|  | *t*_1/2_ | 0.239 (0.026, 1.767) | 0.239 (0.025, 1.806) | 4.97 (0.024, 1.74) | 0.43 (0.025, 1.717) |
|  | σ | 3.442 (0.457, 32.241) | 3.06 (0.395, 28.894) | 0.72 (1.967, 156.775) | 4.04 (0.984, 70.529) |
| OU2_arb&Ma_ | θ (arb&Ma) | 0.009 (-0.009, 0.027) | -0.003 (-0.021, 0.014) | 0.018 (-0.023, 0.061) | 0.018 (-0.015, 0.05) |
|  | θ (Xe) | 0.021 (-0.06, 0.099) | -0.039 (-0.113, 0.038) | -0.382 (-0.567, -0.194) | -0.178 (-0.324, -0.033) |
|  | *t*_1/2_ | 0.249 (0.026, 1.797) | 0.256 (0.026, 1.742) | 8.406 (0.022, 1.768) | 6.763 (0.025, 1.801) |
|  | σ | 3.341 (0.447, 33.007) | 2.884 (0.401, 29.008) | 0.539 (2.436, 214.599) | 0.395 (1.442, 110.551) |
| OU2_arb&Xe_ | θ (arb&Xe) | 0.021 (-0.003, 0.044) | 0.004 (-0.019, 0.027) | 0.018 (-0.039, 0.078) | 0.046 (0.007, 0.087) |
|  | θ (Ma) | -0.003 (-0.028, 0.023) | -0.015 (-0.039, 0.01) | -0.184 (-0.244, -0.124) | -0.137 (-0.18, -0.095) |
|  | *t*_1/2_ | 0.237 (0.025, 1.786) | 0.241 (0.026, 1.802) | 8.039 (0.022, 1.823) | 4.281 (0.024, 1.794) |
|  | σ | 3.464 (0.45, 33.042) | 3.051 (0.401, 29.54) | 0.576 (2.495, 215.309) | 0.539 (1.228, 94.385) |
| OU3 | θ (arb) | 0.021 (-0.005, 0.046) | 0.008 (-0.016, 0.033) | 0.087 (0.035, 0.139) | 0.083 (0.046, 0.121) |
|  | θ (Ma) | -0.003 (-0.029, 0.023) | -0.015 (-0.039, 0.009) | -0.205 (-0.259, -0.154) | -0.143 (-0.181, -0.106) |
|  | θ (Xe) | 0.021 (-0.058, 0.1) | -0.039 (-0.113, 0.036) | -0.372 (-0.539, -0.21) | -0.195 (-0.313, -0.076) |
|  | *t*_1/2_ | 0.237 (0.026, 1.71) | 0.241 (0.026, 1.703) | 4.743 (0.023, 1.699) | 0.412 (0.026, 1.726) |
|  | σ | 3.462 (0.47, 32.218) | 3.019 (0.404, 28.389) | 0.732 (1.972, 151.106) | 4.189 (0.958, 67.361) |

Table S5 (continued).

| Model | Parameter | Teres major fossa CS | Supraspinatus fossa CS | Supraspinatus fossa I-L | Infraspinatus fossa CS |
| --- | --- | --- | --- | --- | --- |
| BM1 | θ | 0.003 (-13.791, 13.91) | -0.028 (-3.762, 3.85) | -0.01 (-3.548, 3.474) | -0.017 (-3.72, 3.608) |
|  | σ* | 3.256 (147.357, 240.944) | 0.242 (10.937, 17.82) | 0.212 (9.536, 15.717) | 0.235 (10.567, 17.414) |
| OU1 | θ (all) | -0.103 (-0.201, -0.005) | 0.009 (-0.01, 0.03) | 0.008 (-0.009, 0.025) | 0.005 (-0.014, 0.024) |
|  | *t*_1/2_ | 0.34 (0.026, 1.712) | 0.245 (0.026, 1.786) | 0.253 (0.026, 1.842) | 0.257 (0.026, 1.75) |
|  | σ | 73.11 (13.952, 1010.113) | 4.316 (0.584, 42.364) | 3.039 (0.406, 30.179) | 3.733 (0.52, 38.991) |
| OU2_foss_ | θ (arb) | 0.242 (0.13, 0.356) | 0.031 (0.002, 0.059) | 0.017 (-0.007, 0.042) | 0.032 (0.004, 0.058) |
|  | θ (foss) | -0.418 (-0.527, -0.313) | -0.01 (-0.038, 0.017) | -0.001 (-0.023, 0.023) | -0.02 (-0.046, 0.006) |
|  | *t*_1/2_ | 0.184 (0.025, 1.756) | 0.223 (0.025, 1.78) | 0.244 (0.025, 1.759) | 0.23 (0.026, 1.789) |
|  | σ | 86.765 (8.709, 645.907) | 4.605 (0.552, 40.683) | 3.124 (0.412, 30.682) | 3.926 (0.491, 35.825) |
| OU2_arb&Ma_ | θ (arb&Ma) | -0.1 (-0.199, -0.003) | 0.007 (-0.013, 0.028) | 0.008 (-0.01, 0.026) | 0.004 (-0.017, 0.024) |
|  | θ (Xe) | -0.156 (-0.597, 0.287) | 0.05 (-0.039, 0.138) | 0.004 (-0.073, 0.08) | 0.013 (-0.071, 0.101) |
|  | *t*_1/2_ | 0.343 (0.026, 1.825) | 0.243 (0.026, 1.788) | 0.253 (0.026, 1.746) | 0.256 (0.025, 1.759) |
|  | σ | 72.518 (13.381, 994.831) | 4.335 (0.562, 41.777) | 3.036 (0.417, 30.26) | 3.739 (0.528, 38.601) |
| OU2_arb&Xe_ | θ (arb&Xe) | 0.205 (0.098, 0.316) | 0.032 (0.007, 0.06) | 0.016 (-0.008, 0.04) | 0.03 (0.005, 0.056) |
|  | θ (Ma) | -0.445 (-0.561, -0.331) | -0.016 (-0.045, 0.012) | -0.001 (-0.026, 0.024) | -0.024 (-0.051, 0.003) |
|  | *t*_1/2_ | 0.179 (0.025, 1.647) | 0.216 (0.025, 1.791) | 0.243 (0.025, 1.747) | 0.227 (0.026, 1.757) |
|  | σ | 90.93 (9.392, 650.001) | 4.676 (0.552, 40.463) | 3.132 (0.416, 30.241) | 3.964 (0.497, 35.559) |
| OU3 | θ (arb) | 0.242 (0.131, 0.353) | 0.031 (0.002, 0.06) | 0.017 (-0.007, 0.042) | 0.032 (0.005, 0.06) |
|  | θ (Ma) | -0.445 (-0.56, -0.333) | -0.016 (-0.046, 0.013) | -0.001 (-0.026, 0.024) | -0.024 (-0.052, 0.004) |
|  | θ (Xe) | -0.156 (-0.506, 0.195) | 0.05 (-0.04, 0.138) | 0.004 (-0.072, 0.081) | 0.013 (-0.07, 0.098) |
|  | *t*_1/2_ | 0.178 (0.025, 1.663) | 0.216 (0.025, 1.72) | 0.243 (0.026, 1.699) | 0.227 (0.025, 1.762) |
|  | σ | 88.035 (9.118, 624.386) | 4.67 (0.543, 39.753) | 3.13 (0.433, 29.733) | 3.959 (0.502, 35.682) |

Table S5 (continued).

| Model | Parameter | Infraspinatus fossa I-L | Subscapularis fossa CS | Subscapularis fossa I-L |
| --- | --- | --- | --- | --- |
| BM1 | θ | -0.017 (-3.584, 3.477) | -0.021 (-3.723, 3.792) | -0.014 (-3.471, 3.54) |
|  | σ* | 0.207 (9.238, 15.344) | 0.237 (10.62, 17.551) | 0.212 (9.468, 15.577) |
| OU1 | θ (all) | 0.005 (-0.012, 0.023) | 0.006 (-0.013, 0.025) | 0.007 (-0.011, 0.024) |
|  | *t*_1/2_ | 0.274 (0.025, 1.806) | 0.252 (0.026, 1.832) | 0.259 (0.026, 1.804) |
|  | σ | 2.924 (0.429, 32.113) | 3.912 (0.524, 40.406) | 3.014 (0.412, 31.399) |
| OU2_foss_ | θ (arb) | 0.017 (-0.007, 0.043) | 0.031 (0.003, 0.059) | 0.016 (-0.009, 0.041) |
|  | θ (foss) | -0.005 (-0.029, 0.018) | -0.016 (-0.042, 0.009) | -0.002 (-0.026, 0.022) |
|  | *t*_1/2_ | 0.263 (0.025, 1.784) | 0.227 (0.025, 1.7) | 0.25 (0.025, 1.773) |
|  | σ | 3.011 (0.445, 31.608) | 4.144 (0.528, 37.388) | 3.094 (0.431, 30.862) |
| OU2_arb&Ma_ | θ (arb&Ma) | 0.005 (-0.013, 0.022) | 0.005 (-0.014, 0.025) | 0.007 (-0.011, 0.024) |
|  | θ (Xe) | 0.017 (-0.064, 0.095) | 0.028 (-0.059, 0.118) | 0.01 (-0.067, 0.086) |
|  | *t*_1/2_ | 0.274 (0.026, 1.786) | 0.251 (0.026, 1.834) | 0.259 (0.026, 1.798) |
|  | σ | 2.929 (0.43, 32.063) | 3.925 (0.522, 39.428) | 3.016 (0.427, 30.558) |
| OU2_arb&Xe_ | θ (arb&Xe) | 0.017 (-0.007, 0.04) | 0.031 (0.005, 0.057) | 0.016 (-0.008, 0.039) |
|  | θ (Ma) | -0.007 (-0.033, 0.017) | -0.021 (-0.047, 0.007) | -0.003 (-0.027, 0.023) |
|  | *t*_1/2_ | 0.261 (0.026, 1.745) | 0.222 (0.025, 1.743) | 0.249 (0.026, 1.763) |
|  | σ | 3.029 (0.444, 30.624) | 4.189 (0.521, 36.808) | 3.106 (0.431, 30.555) |
| OU3 | θ (arb) | 0.017 (-0.009, 0.042) | 0.031 (0.004, 0.059) | 0.016 (-0.009, 0.04) |
|  | θ (Ma) | -0.007 (-0.032, 0.018) | -0.021 (-0.049, 0.007) | -0.003 (-0.028, 0.023) |
|  | θ (Xe) | 0.017 (-0.061, 0.097) | 0.028 (-0.057, 0.114) | 0.01 (-0.065, 0.088) |
|  | *t*_1/2_ | 0.261 (0.026, 1.756) | 0.222 (0.026, 1.693) | 0.249 (0.025, 1.729) |
|  | σ | 3.03 (0.435, 30.818) | 4.189 (0.522, 36.851) | 3.106 (0.43, 30.09) |

**Table S6. Estimated model parameters and their confidence intervals concerning the femoral dataset.** EL = effective length, CS = centroid size, apD = anteroposterior diameter, mlD = mediolateral diameter, W = width, I-L = in-lever. Asterisks indicates empirical parameter that always fell outside of its confidence interval.

| Model | Parameter | Head CS | Midshaft apD | Midshaft mlD | Medial condyle W |
| --- | --- | --- | --- | --- | --- |
| BM1 | θ | 0.036 (-3.037, 3.194) | 0.014 (-3.577, 3.577) | 0.043 (-3.311, 3.359) | 0 (-3.941, 3.738) |
|  | σ* | 0.167 (7.45, 12.396) | 0.21 (9.448, 15.554) | 0.187 (8.385, 13.789) | 0.253 (11.243, 18.649) |
| OU1 | θ (all) | -0.006 (-0.023, 0.011) | -0.014 (-0.036, 0.008) | 0.001 (-0.022, 0.023) | 0.022 (0.003, 0.041) |
|  | *t*_1/2_ | 0.317 (0.026, 1.941) | 0.782 (0.025, 1.902) | 0.735 (0.025, 1.932) | 0.212 (0.025, 1.909) |
|  | σ | 2.237 (0.362, 28.014) | 1.662 (0.669, 51.566) | 1.807 (0.687, 53.481) | 4.764 (0.509, 40.604) |
| OU2_foss_ | θ (arb) | 0.02 (0, 0.039) | 0.02 (-0.008, 0.047) | 0.024 (-0.003, 0.052) | 0.017 (-0.008, 0.041) |
|  | θ (foss) | -0.046 (-0.07, -0.02) | -0.067 (-0.102, -0.033) | -0.035 (-0.071, 0) | 0.031 (0, 0.062) |
|  | *t*_1/2_ | 0.241 (0.025, 1.799) | 0.594 (0.026, 1.781) | 0.683 (0.026, 1.853) | 0.228 (0.026, 1.859) |
|  | σ | 2.607 (0.331, 25.46) | 1.943 (0.626, 45.947) | 1.845 (0.662, 50.387) | 4.412 (0.524, 39.964) |
| OU2_arb&Ma_ | θ (arb&Ma) | -0.006 (-0.022, 0.012) | -0.016 (-0.039, 0.007) | -0.002 (-0.025, 0.022) | 0.018 (-0.001, 0.038) |
|  | θ (Xe) | -0.013 (-0.088, 0.061) | 0.04 (-0.061, 0.141) | 0.048 (-0.05, 0.151) | 0.096 (0.008, 0.184) |
|  | *t*_1/2_ | 0.318 (0.026, 1.876) | 0.77 (0.026, 1.839) | 0.718 (0.026, 1.939) | 0.205 (0.025, 1.839) |
|  | σ | 2.232 (0.361, 28.094) | 1.67 (0.673, 50.493) | 1.836 (0.658, 52.145) | 4.815 (0.52, 40.001) |
| OU2_arb&Xe_ | θ (arb&Xe) | 0.017 (-0.002, 0.036) | 0.022 (-0.004, 0.046) | 0.026 (-0.001, 0.052) | 0.023 (-0.001, 0.047) |
|  | θ (Ma) | -0.05 (-0.076, -0.025) | -0.083 (-0.119, -0.047) | -0.047 (-0.085, -0.01) | 0.022 (-0.012, 0.054) |
|  | *t*_1/2_ | 0.233 (0.026, 1.872) | 0.559 (0.025, 1.785) | 0.666 (0.026, 1.837) | 0.212 (0.025, 1.845) |
|  | σ | 2.688 (0.328, 24.575) | 1.973 (0.593, 43.951) | 1.849 (0.641, 48.654) | 4.779 (0.543, 39.71) |
| OU3 | θ (arb) | 0.02 (0, 0.04) | 0.02 (-0.007, 0.047) | 0.024 (-0.005, 0.052) | 0.017 (-0.008, 0.043) |
|  | θ (Ma) | -0.05 (-0.077, -0.025) | -0.083 (-0.116, -0.048) | -0.047 (-0.084, -0.01) | 0.022 (-0.01, 0.055) |
|  | θ (Xe) | -0.013 (-0.084, 0.054) | 0.04 (-0.054, 0.136) | 0.048 (-0.053, 0.146) | 0.096 (0.012, 0.185) |
|  | *t*_1/2_ | 0.233 (0.026, 1.759) | 0.558 (0.026, 1.822) | 0.658 (0.026, 1.708) | 0.211 (0.026, 1.812) |
|  | σ | 2.673 (0.336, 24.579) | 1.971 (0.584, 43.104) | 1.866 (0.672, 48.18) | 4.674 (0.518, 39.033) |

Table S6 (continued).

| Model | Parameter | Lateral condyle W | Patellar groove W | Lesser trochanter I-L |
| --- | --- | --- | --- | --- |
| BM1 | θ | 0.022 (-2.773, 2.895) | 0.053 (-3.849, 4.093) | -0.025 (-4.259, 4.233) |
|  | σ* | 0.139 (6.193, 10.199) | 0.272 (12.232, 20.245) | 0.299 (13.343, 22.017) |
| OU1 | θ (all) | 0.02 (-0.002, 0.042) | -0.016 (-0.04, 0.009) | -0.012 (-0.034, 0.01) |
|  | *t*_1/2_ | 4.045 (0.025, 1.944) | 0.561 (0.025, 1.899) | 0.316 (0.025, 1.907) |
|  | σ | 0.314 (0.642, 51.078) | 2.744 (0.783, 62.898) | 3.96 (0.633, 50.435) |
| OU2_foss_ | θ (arb) | 0.048 (0.024, 0.073) | 0.032 (0.004, 0.061) | 0.012 (-0.014, 0.039) |
|  | θ (foss) | -0.059 (-0.089, -0.028) | -0.091 (-0.125, -0.056) | -0.049 (-0.082, -0.013) |
|  | *t*_1/2_ | 1.272 (0.026, 1.825) | 0.435 (0.025, 1.87) | 0.265 (0.026, 1.858) |
|  | σ | 0.764 (0.519, 38.402) | 2.87 (0.653, 49.623) | 4.446 (0.627, 47.746) |
| OU2_arb&Ma_ | θ (arb&Ma) | 0.023 (0.001, 0.046) | -0.019 (-0.044, 0.006) | -0.012 (-0.034, 0.01) |
|  | θ (Xe) | -0.012 (-0.109, 0.086) | 0.041 (-0.068, 0.153) | -0.007 (-0.106, 0.092) |
|  | *t*_1/2_ | 4.071 (0.026, 1.913) | 0.549 (0.025, 1.92) | 0.316 (0.026, 1.848) |
|  | σ | 0.312 (0.636, 50.465) | 2.776 (0.767, 60.567) | 3.963 (0.658, 49.347) |
| OU2_arb&Xe_ | θ (arb&Xe) | 0.043 (0.019, 0.067) | 0.033 (0.006, 0.059) | 0.011 (-0.016, 0.038) |
|  | θ (Ma) | -0.066 (-0.099, -0.033) | -0.11 (-0.146, -0.074) | -0.055 (-0.092, -0.018) |
|  | *t*_1/2_ | 1.234 (0.025, 1.762) | 0.407 (0.025, 1.866) | 0.258 (0.026, 1.766) |
|  | σ | 0.791 (0.534, 39.245) | 2.87 (0.604, 45.997) | 4.546 (0.645, 46.294) |
| OU3 | θ (arb) | 0.048 (0.023, 0.072) | 0.032 (0.004, 0.059) | 0.012 (-0.015, 0.04) |
|  | θ (Ma) | -0.066 (-0.099, -0.034) | -0.11 (-0.145, -0.074) | -0.055 (-0.09, -0.018) |
|  | θ (Xe) | -0.013 (-0.101, 0.072) | 0.041 (-0.056, 0.131) | -0.007 (-0.101, 0.092) |
|  | *t*_1/2_ | 1.253 (0.025, 1.758) | 0.406 (0.026, 1.774) | 0.258 (0.026, 1.741) |
|  | σ | 0.768 (0.521, 38.422) | 2.874 (0.616, 45.761) | 4.542 (0.632, 46.345) |

**Table S7. Specimens used to assess the intraspecific variation for the simulation study.** See Table S1 for abbreviations of collection names.

| Skeletal element | Species | Collection | Catalog No. |
| --- | --- | --- | --- |
| Scapula | *Tamias striatus* | FM | 108316 |
|  |  |  | 156745 |
|  |  |  | 159797 |
|  |  |  | 163171 |
|  |  |  | 167171 |
|  |  |  | 167177 |
|  |  |  | 199957 |
|  |  |  | 199958 |
|  |  | UMMZ | 67521 |
|  |  |  | 67523 |
|  |  |  | 81052 |
|  |  |  | 81158 |
|  |  |  | 101049 |
|  |  |  | 124620 |
|  |  |  | 161416 |
|  |  |  | 161419 |
|  |  |  | 176201 |
|  |  |  | 176217 |
|  |  | ZMB | Mam_88869* |
|  | *Sciurus carolinensis* | FM | 140290 |
|  |  |  | 154677 |
|  |  |  | 154680 |
|  |  |  | 156872 |
|  |  |  | 156880 |
|  |  |  | 187476 |
|  |  |  | 187479 |
|  |  | SNMNH | 548056 |
|  |  | UMMZ | 65254 |
|  |  |  | 65813 |
|  |  |  | 75060 |
|  |  |  | 81726 |
|  |  |  | 101807 |
|  |  |  | 115794 |
|  |  |  | 115795 |
|  |  |  | 118464 |
|  |  |  | 123677 |
|  |  |  | 165019 |
|  |  |  | 165023 |
|  |  |  | 81824* |
|  | *Marmota monax* | FM | 156742 |
|  |  |  | 178041 |
|  |  |  | 178043 |
|  |  | SNMNH | 20765 |
|  |  |  | 191374 |
|  |  |  | 258735 |
|  |  |  | 259345 |
|  |  |  | 349710 |
|  |  |  | 397379 |
|  |  |  | 506225 |
|  |  |  | 514748 |
|  |  |  | 567646 |
|  |  |  | A35137 |
|  |  |  | A807 |
|  |  | UMMZ | 53654 |
|  |  |  | 59889 |
|  |  |  | 168358 |
|  |  |  | 170476 |
|  |  |  | 170478 |
|  |  |  | 173712 |
|  |  |  | 166225* |
| Femur | *Tamiasciurus hudsonicus* | FM | 20353 |
|  |  |  | 20354 |
|  |  |  | 43950 |
|  |  |  | 122027 |
|  |  |  | 163324 |
|  |  |  | 163326 |
|  |  |  | 173670 |
|  |  | SNMNH | 7258 |
|  |  | UMMZ | 59376 |
|  |  |  | 61951 |
|  |  |  | 67531 |
|  |  |  | 75059 |
|  |  |  | 81735 |
|  |  |  | 83623 |
|  |  |  | 95919 |
|  |  |  | 95920 |
|  |  |  | 11423* |
|  |  | zmb | 95202 |
|  | *Sciurus carolinensis* | SNMNH | 347937 |
|  |  |  | 347939 |
|  |  |  | 347948 |
|  |  |  | 396337 |
|  |  |  | 397048 |
|  |  |  | 397178 |
|  |  |  | 397179 |
|  |  |  | 397210 |
|  |  |  | 398408 |
|  |  |  | 398504 |
|  |  |  | 500984 |
|  |  |  | 500987 |
|  |  |  | 527934 |
|  |  |  | 527938 |
|  |  |  | 528049 |
|  |  |  | 548048 |
|  |  |  | 548051 |
|  |  |  | 570626 |
|  |  |  | 570628 |
|  |  |  | 599264 |
|  |  |  | 599267 |
|  |  |  | 599273 |
|  |  |  | 599277 |
|  |  |  | 548056* |
|  | *Marmota monax* | FM | 156742 |
|  |  |  | 178041 |
|  |  | SNMNH | 20765 |
|  |  |  | 191374 |
|  |  |  | 258735 |
|  |  |  | 259345 |
|  |  |  | 349710 |
|  |  |  | 396282 |
|  |  |  | 397378 |
|  |  |  | 397379 |
|  |  |  | 506225 |
|  |  |  | 514748 |
|  |  |  | 567646 |
|  |  |  | A35137 |
|  |  | UMMZ | 53654 |
|  |  |  | 59889 |
|  |  |  | 168358 |
|  |  |  | 170476 |
|  |  |  | 170478 |
|  |  |  | 170527 |
|  |  |  | 173712 |
|  |  |  | 178655 |
|  |  | ZMB | 12055* |

* Specimen used in the interspecific dataset (see Tables S1 & S2)

**Table S8. Standard deviation for the traits of the specimens used to assess the intraspecific variation for the simulation study.** See Tables S5 & S6 for trait abbreviations.

| Skeletal element | Trait | Standard deviation [mm] | | |
| --- | --- | --- | --- | --- |
| Scapula |  | *Marmota monax* | *Sciurus carolinensis* | *Tamias striatus* |
|  | Scapula EL | 3.852 | 2.078 | 0.985 |
|  | Glenoid cavity CS_ca_ | 1.832 | 0.666 | 0.406 |
|  | Coracoid process CS | 6.777 | 3.305 | 1.650 |
|  | Coracoid process I-L_ml_ | 0.723 | 0.304 | 0.176 |
|  | Teres major fossa CS | 6.857 | 3.154 | 2.787 |
|  | Supraspinatus fossa CS | 13.242 | 6.966 | 2.951 |
|  | Supraspinatus fossa I-L | 2.088 | 1.113 | 0.527 |
|  | Infraspinatus fossa CS | 18.434 | 9.662 | 4.367 |
|  | Infraspinatus fossa I-L | 2.215 | 1.233 | 0.604 |
|  | Subscapularis fossa CS | 31.212 | 16.542 | 7.262 |
|  | Subscapularis fossa I-L | 2.102 | 1.153 | 0.559 |
| Femur |  | *Marmota monax* | *Sciurus carolinensis* | *Tamiasciurus hudsonicus* |
|  | Head CS | 1.650 | 0.922 | 0.525 |
|  | Midshaft apD | 0.527 | 0.303 | 0.215 |
|  | Midshaft mlD | 0.777 | 0.360 | 0.210 |
|  | Medial condyle W | 0.578 | 0.267 | 0.154 |
|  | Lateral condyle W | 0.541 | 0.375 | 0.244 |
|  | Patellar groove W | 0.399 | 0.257 | 0.185 |
|  | Lesser Trochanter I-L | 0.897 | 0.470 | 0.401 |

**Literature**

Angermann, R. (1963). Zur Ökologie und Biologie des Baumschläfers, *Dryomys nitedula* (Pallas, 1779) in der Waldsteppenzone. *Acta Theriologica, 7*(18), 333-367.

Bertolino, S., Cordero, N., & Currado, I. (2003). Home ranges and habitat use of the garden dormouse (*Eliomys quercinus*) in a mountain habitat in summer. *Acta Zoologica Academiae Scientiarum Hungaricae, 49*, 11-18.

Carraway, L. N., & Verts, B. (1993). *Aplodontia rufa*. *Mammalian Species Archive, 431*, 1-10.

Csorba, A. (2003). Influence of body weight on hibernation of the common dormouse (*Muscardinus avellanarius*). *Acta Zoologica Academiae Scientiarum Hungaricae, 49*, 39-44.

Fietz, J., Pflug, M., Schlund, W., & Tataruch, F. (2005). Influences of the feeding ecology on body mass and possible implications for reproduction in the edible dormouse (*Glis glis*). *Journal of Comparative Physiology. B: Biochemical, Systemic, and Environmental Physiology, 175*(1), 45-55. doi:10.1007/s00360-004-0461-1

Hayssen, V. (2008). Patterns of body and tail length and body mass in Sciuridae. *Journal of Mammalogy, 89*(4), 852-873. doi:10.1644/07-mamm-a-217.1

Hedges, S. B., Marin, J., Suleski, M., Paymer, M., & Kumar, S. (2015). Tree of life reveals clock-like speciation and diversification. *Molecular Biology and Evolution, 32*(4), 835-845. doi:10.1093/molbev/msv037

Holden, M. E., & Levine, R. S. (2009). Chapter 9. Systematic revision of sub-saharan African dormice (Rodentia: Gliridae: *Graphiurus*) Part II: description of a wew species of *Graphiurus* from the central Congo Basin, including morphological and ecological niche comparisons with *G. crassicaudatus* and *G. lorraineus*. *Bulletin of the American Museum of Natural History, 331*(1), 314-355. doi:10.1206/582-9.1

Kastenmayer, R. J., Moak, H. B., Jeffress, E. J., & Elkins, W. R. (2010). Management and care of African dormice (*Graphiurus kelleni*). *J Am Assoc Lab Anim Sci, 49*(2), 173-176.

Kingdon, J. (2015). *The Kingdon field guide to African mammals*. London, UK: Bloomsbury Publishing.

Kryštufek, B., & Vohralík, V. (2012). Taxonomic revision of the Palaearctic rodents (Rodentia). Sciuridae: Xerinae 1 (*Eutamias* and *Spermophilus*). *Lynx, n. s. (Praha), 43*(1-2), 17-111.

Nowak, R. M. (1999). *Walker's mammals of the world* (6 ed. Vol. 2). Baltimore, MD: The John Hopkins University Press.

Shehab, A. H., Asaad, M., & Mamkhair, I. (2009). Morphology and distribution of the Asian Garden Dormouse, *Eliomys melanurus* Wagner, 1840, in Syria. *Zoology in the Middle East, 48*(1), 3-12. doi:10.1080/09397140.2009.10638360

Smith, A. T., Xie, Y., Hoffmann, R. S., Lunde, D., MacKinnon, J., Wilson, D. E., . . . Gemma, F. (2010). *A guide to the mammals of China*. Princeton, NJ: Princeton University Press.

Thorington Jr., R. W., Koprowski, J. L., Steele, M. A., & Whatton, J. F. (2012). *Squirrels of the world*. Baltimore, MD: John Hopkins University Press.

Webb, P. I., & Skinner, J. D. (1996). Summer torpor in African woodland dormice *Graphiurus murinus* (Myoxidae: Graphiurinae). *Journal of Comparative Physiology B, 166*(5), 325-330. doi:10.1007/bf02439919

Wölfer, J., Amson, E., Arnold, P., Botton-Divet, L., Fabre, A. C., van Heteren, A. H., & Nyakatura, J. A. (2019). Femoral morphology of sciuromorph rodents in light of scaling and locomotor ecology. *Journal of Anatomy*. doi:doi: 10.1111/joa.12980

Wölfer, J., Arnold, P., & Nyakatura, J. A. (2019). Effects of scaling and locomotor ecology suggest a complex evolution of the scapular morphology in sciuromorph rodents. *Biological Journal of the Linnean Society*. doi:10.1093/biolinnean/blz042

Zelditch, M. L., Li, J., Tran, L. A., & Swiderski, D. L. (2015). Relationships of diversity, disparity, and their evolutionary rates in squirrels (Sciuridae). *Evolution, 69*(5), 1284-1300. doi:10.1111/evo.12642
